# Sponge-Derived 24-Homoscalaranes as Potent Anti-Inflammatory Agents

**DOI:** 10.3390/md18090434

**Published:** 2020-08-19

**Authors:** Bo-Rong Peng, Kuei-Hung Lai, Yu-Chia Chang, You-Ying Chen, Jui-Hsin Su, Yusheng M. Huang, Po-Jen Chen, Steve Sheng-Fa Yu, Chang-Yih Duh, Ping-Jyun Sung

**Affiliations:** 1 Doctoral Degree Program in Marine Biotechnology, National Sun Yat-Sen University, Kaohsiung 804201, Taiwan; pengpojung@gmail.com; 2 Doctoral Degree Program in Marine Biotechnology, Academia Sinica, Taipei 115201, Taiwan; 3 National Museum of Marine Biology and Aquarium, Pingtung 944401, Taiwan; zoeblack0108@gmail.com (Y.-Y.C.); x2219@nmmba.gov.tw (J.-H.S.); 4 Graduate Institute of Pharmacognosy, College of Pharmacy, Taipei Medical University, Taipei 110301, Taiwan; mos19880822@gmail.com; 5 Research Center for Chinese Herbal Medicine, College of Human Ecology, Chang Gung University of Science and Technology, Taoyuan 333324, Taiwan; jay0404@gmail.com; 6 Department of Marine Biotechnology and Resources, National Sun Yat-Sen University, Kaohsiung 804201, Taiwan; 7 Graduate Institute of Marine Biology, National Dong Hwa University, Pingtung 944401, Taiwan; 8 Department of Marine Recreation, National Penghu University of Science and Technology, Penghu 880011, Taiwan; yusheng@npu.edu.tw; 9 Department of Cosmetic Science, Providence University, Taichung 433303, Taiwan; litlep@hotmail.com; 10 Institute of Chemistry, Academia Sinica, Taipei 115201, Taiwan; 11 Chinese Medicine Research and Development Center, China Medical University Hospital, Taichung 404333, Taiwan; 12 Graduate Institute of Natural Products, Kaohsiung Medical University, Kaohsiung 807377, Taiwan

**Keywords:** 24-homoscalarane, sesterterpenoid, lendenfeldarane, *Lendenfeldia*, anti-neutrophilic inflammation, superoxide anion, NADPH oxidase, elastase release

## Abstract

Scalarane-type sesterterpenoids are known for their therapeutic potential in cancer treatments. However, the anti-inflammatory properties of this class of metabolites remain elusive. Our current work aimed to investigate the anti-inflammatory scalaranes from marine sponge *Lendenfeldia* sp., resulting in the isolation of six new 24-homoscalaranes, lendenfeldaranes E–J (**1**–**6**). The structures of the new metabolites were determined by extensive spectroscopic analyses, and the absolute configuration of **1** was established by electronic circular dichroism (ECD) calculations. Compounds **2** and **3** were discovered to individually reduce the generation of superoxide anions, and compound **1** displayed an inhibitor effect on the release of elastase. These three compounds were proven to be the first anti-neutrophilic scalaranes.

## 1. Introduction

**Figure 1:**
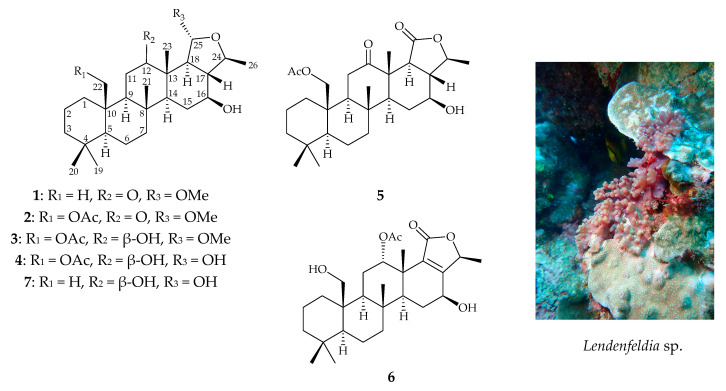
Structures of lendenfeldaranes E–J (**1**–**6**) and felixin E (**7**) and a picture of *Lendenfeldia* sp.

The marine sponges of genus *Lendenfeldia* have been studied for decades since first being reported in 1982 [1]. Further investigation of this genus revealed more than 50 compounds categorized into scalarane-type sesterterpenoids [1,2,3,4,5], other types of sesterterpenoids [4], amino acids [6], steroids [7,8], iminosugars [9], naphthalenes [10], lipid [10,11], and diphenyl ethers [12]. Our group has extensively studied scalarane-type sesterterpenoids over the past few years and has found that they demonstrate a wide structural diversity [5,13,14,15,16]. This class of compounds possesses a pentamethyl-D-homoandrostane skeleton. Alkylated scalaranes are usually known as homoscalarane, exhibiting methylation at C-20 or C-24. In the current report, we summarize a series of structural classification for scalaranes from the *Lendenfeldia* sp. sponge. In detail, a normal 25-carbon scalarane represents the basic type of scalarane, while the 26-carbon ones can be further sorted into tetra- and pentacyclic homoscalarane groups. Nor-homoscalaranes were characterized with a missing methyl at position 18 and bishomoscalaranes are defined for the scalaranes with a pair of methylations at both C-20 and C-24. Additionally, it is noteworthy that most of them present a different oxidation in positions C-12, C-16, C-22, C-24, and C-25 [17]. The biological properties of scalarane-type sesterterpenoids were extensively studied with special emphasis on cytotoxic and anti-proliferative properties [5]. For instance, the scalaranes isolated from sponges of *Hyrtios*, *Hippospongia*, *Lendenfeldia*, *Phyllospongia*, and *Psammocinia* genus were examined to show potent cytotoxicity against A498, ACHN, MIA-paca, PANC-1, CV-1, molt-4, K562, DLD-1, HCT-116, and T-47D cancer cell lines at low concentrations (< 4 μM) [5,15,18]. The previous pharmacological studies on scalaranes have also revealed several possible anti-proliferative mechanisms, including the inhibition of Hsp90 and topoisomerase II [16], and the binding of DNA [19]. In addition, these sesterterpenoids were also reported to exhibit other pharmacological activities, such as anti-microbial, anti-fungal, anti-viral, and so on [16]. However, only few studies have explored the anti-inflammatory activity of this class of metabolites. A sponge-derived scalarane, named deacetylphylloketa, was reported to exhibit anti-inflammatory activity by regulating the expression levels of pro-inflammatory factors (TNF-α, IL-6, and IL-1β) and anti-inflammatory factors (Nrf-2 and HO-1). It could downregulate the expressions of iNOS and COX-2, as well as attenuate nuclear translocation of NF-κB [20]. Recently, we focused our ongoing studies on a marine sponge identified as *Lendenfeldia* sp. From the result of our studies on this species, we report herein the isolation, structural determination, and bioactivity of six new 24-homoscalaranes, lendenfeldaranes E–J (**1**–**6**) (Figure 1). Moreover, the extensive biological screening suggested the isolates significantly inhibited superoxide anion generation and elastase release in neutrophils responding to *N*-formyl-methionyl-leucyl-phenylalanine (fMLF).

## 2. Results and Discussion

Specimens of the marine sponge *Lendenfeldia* sp. were collected by hand by self-contained underwater breathing apparatus (scuba) diving off the coast of Southern Taiwan in 2012, and stored frozen at −20 °C until extraction. The frozen sponge was minced and extracted with ethyl acetate (EtOAc). The fractionation of the EtOAc-soluble extract led to the production of 11 fractions A–K. Fractions I and J were further purified by normal-phase and reversed-phase HPLC to afford scalaranes **1**–**6**.

Lendenfeldarane E (**1**), isolated as an amorphous powder, has a molecular formula of C_27_H_44_O_4_ as determined from its (+)-HRESIMS at *m*/*z* 455.31340 (calcd. for C_27_H_44_O_4_ + Na, 455.31318) implying 6 degrees of unsaturation. The IR spectrum showed absorptions for OH (3438 cm^−1^) and C=O (1701 cm^−1^) functionalities. The ^1^H NMR data (Table 1) demonstrated five tertiary methyls at δ_H_ 0.82, 0.85, 0.86, 1.07, and 1.36 (each 3H × s); one secondary methyl at δ_H_ 1.40 (3H, d, *J* = 6.0 Hz); one methoxy at δ_H_ 3.30 (3H, s); as well as three oxymethine protons at 3.43 (1H, ddd, *J* = 10.4, 10.4, 4.4 Hz), 3.84 (1H, qd, *J* = 6.0, 2.4 Hz), and 5.35 (1H, d, *J* = 4.0 Hz). The ^13^C NMR (Table 1), heteronuclear single quantum correlation (HSQC), and distortionless enhancement by polarization transfer (DEPT) spectra revealed in total 27 carbon signals including a few oxygenated ones, such as a ketone (δ_C_ 215.3), a ketal carbon (δ_C_ 104.8), and two oxymethine carbons (δ_C_ 74.8 and 78.7).

**Figure 2:**
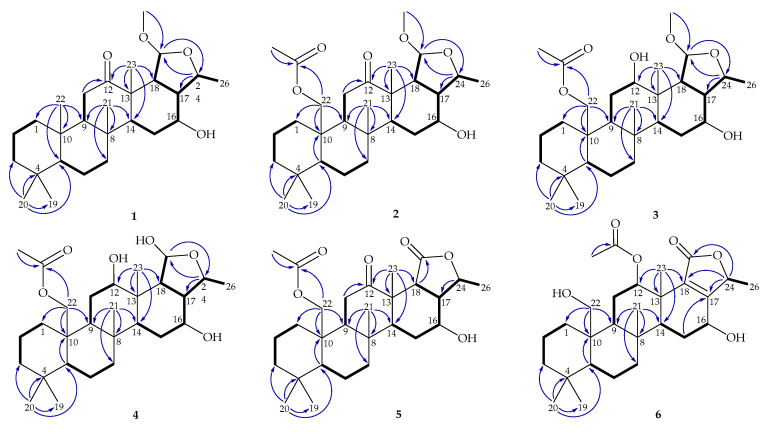
The COSY (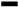) and HMBC (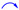) of **1**–**6**.

**Figure 3:**
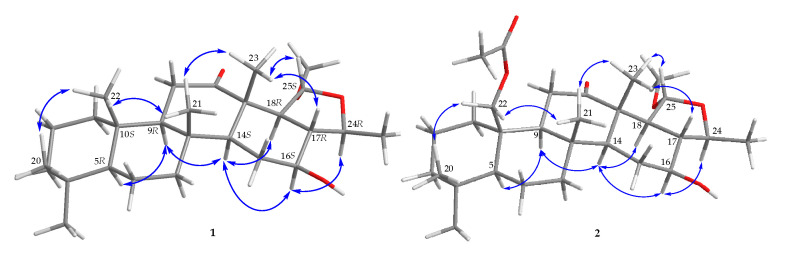
The selected NOESY correlations (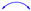) of **1** and **2**.

**Figure 4:**
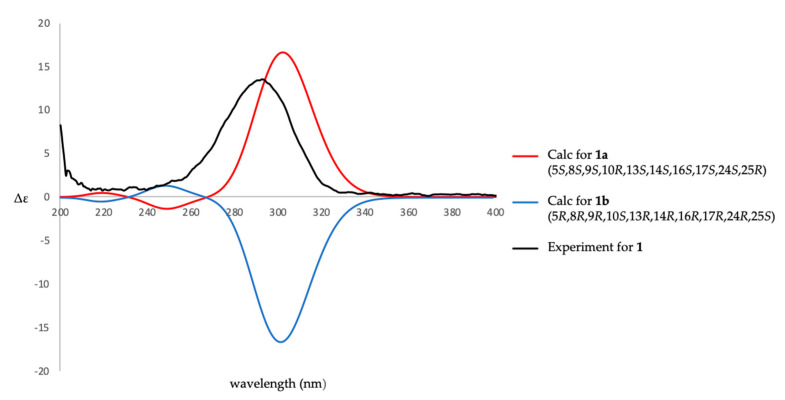
Calculated and experimental electronic circular dichroism (ECD) spectra of **1**.

After the detailed analysis of above NMR data, one degree of unsaturation (ketone) was found to form a part of total. Then the rest of the five unsaturated degrees obscured a pentacyclic homoscalarane. This inference can be further confirmed from the heteronuclear multiple bond correlation (HMBC) (Figure 2) of H_3_-19 to C-3, C-4, C-5, and C-20; H_3_-21 to C-7, C-8, C-9, and C-14; H_3_-22 to C-1, C-5, C-9, and C-10; H_3_-23 to C-12, C-13, C-14, and C-18; H-24 to C-16, C-17, C-18, and C-25; H_3_-26 to C-17 and C-24; 25-methoxy to C-25, and further confirmed by the ^1^H–^1^H correlation spectroscopy (COSY) (Figure 2). Thus, it indicated that compound **1** is a 6/6/6/6/5 pentacyclic scalarane sesterterpene, having a 2-methoxy-5-methyltetrahydrofuran. A detailed analysis of these NMR data with those of a known metabolite, felixin E (**7**) [14], suggested that the structure of **1** is closely related to that of **7**, with the only difference being an α-hydroxy group at C-25 in **7** replaced by an α-methoxy group in **1 [14]**. All naturally occurring scalarane sesterterpenoids displayed the H-5 *trans* to Me-22, assigned as α- and β-orientation, respectively [17]. Then, the relative stereochemistry of **1** was established by nuclear Overhauser effect spectroscopy (NOESY) spectral analysis. The NOESY experimental data (Figure 3) demonstrated the correlations H_3_-22/H_3_-20, H_3_-22/H_3_-21, H_3_-21/ H_3_-23, H_3_-23/H-17, H_3_-23/H-25, H-5/H-9, H-9/H-14, H-14/16, H-14/H-18, and H-16/H-24, supporting the β-Me-20, β-Me-21, β-Me-23, β-H-17, α-OMe-25, α-H-9, α-H-14, β-OH-16, α-H-18, and β-Me-26 assignments. The aforementioned results enabled the establishment of the relative configuration of **1**. Based on the above findings; the configurations of stereogenic carbons of **1** were determined as 5S*, 8R*,9S*,10R*,13S*,14S*,16S*,17S*,18S*,24S*, and 25R*. To further determine the absolute configuration of **1**, the electronic circular dichroism (ECD) calculations for the enantiomers of **1**, including **1a** (5*S*,8*R*,9*S*,10*R*,13*S*,14*S*,16*S*,17*S*,18*S*,24*S*, and 25*R*) and **1b** (5*R*,8*S*,9*R*,10*S*,13*R*,14*R*,16*R*,17*R*, 18*R*,24*R*, and 25*S*) were performed using the method at the B3LYP/6-31þG* level with Gaussian 9.0 software (Figure 4). By comparison of the experimental and calculated ECD spectra, the result of compound **1** was in good agreement with that of **1a**, inferring the 5*S*,8*R*,9*S*,10*R*,13*S*,14*S*,16*S*,17*S*,18*S*, 24*S*, and 25*R* configurations. Hence, the structure, including the absolute configuration of lendenfeldarane E (**1**) was unambiguously assigned as shown in Figure 1 and Figure 3 (Supplementary Materials, Figures S1–S8).

Lendenfeldarane F (**2**), an amorphous powder, showed the molecular formula C_29_H_46_O_6_ determined by (+)-HRESIMS and ^13^C NMR data, implying seven unsaturated degrees. The IR spectra indicated the presence of OH (3451 cm^−1^), ester carbonyl (1731 cm^−1^), and C=O (1700 cm^−1^) functional groups. The ^1^H, ^13^C, DEPT, and HSQC spectra (Table 1) displayed seven methyls, one oxymethylene, two oxymethines, one ester carbonyl, one ketone, and one ketal carbon, representing two unsaturated calculations. Thus, the above NMR data and the rest of the five unsaturated degrees of **2** implied a pentacyclic homoscalarane. The NMR data of **2** resembled those of **1** with the exception of an additional oxymethylene signal (δ_C_ 64.6; δ_H_ 4.19, 1H, dd, *J* = 12.4, 1.6 Hz; 4.65, 1H, d, *J* = 12.4 Hz, CH_2_-22), and an acetoxy group (δ_H_ 2.04, 3H, s; δ_C_ 170.7, C; 21.1, CH_3_). An ethyl acetate substitution at position 10 can be deduced by HMBC cross-peaks from H_2_-22 to C-1, C-5, C-9, C-10, and acetate carbonyl. The stereochemical configuration was identical to that of other scalarane sesterterpenes based on the NOESY cross-correlations at H_3_-22/H_3_-20, H_2_-22/H_3_-21, H_3_-21/H_3_-23, H_3_-23/H-25, H-5/H-9, H-9/H-14, H-14/16, H-14/H-18, and H-16/H-24 (Figure 3). Based on the above findings, the configurations of stereogenic carbons of **2** were determined to be 5S*,8R*,9S*,10R*,13S*,14S*,16S*, 17S*,18S*,24S*, and 25R*. As 24-homoscalaranes **2**–**6** were isolated along with **1** from the same target organism, it is reasonable on biogenetic grounds to assume that **2**–**6** have the same absolute configurations as that of **1**. Therefore, the configurations of stereogenic centers of **2** were determined as 5*S*,8*R*,9*S*,10*R*,13*S*,14*S*,16*S*,17*S*,18*S*,24*S*, and 25*R* (Supplementary Materials, Figures S9–S16).

Compound **3** (lendenfeldarane G) was obtained as an amorphous powder. Its molecular formula was determined to be C_29_H_48_O_6_ by (+)-HRESIMS with six degrees of unsaturation. The IR spectra indicated the presence of OH (3462 cm^−1^) and ester carbonyl (1729 cm^−1^) functionalities. 1D and 2D NMR data disclosed a 6/6/6/6/5 pentacyclic skeleton, which was closely related to compound **2**. The only difference between these two compounds was a reductive substitution at C-12 in **3**. Comparing the ^1^H and ^13^C NMR data (Table 2) of **3** with those of **2** showed an extra oxymethine signal (δ_C_ 72.0; δ_H_ 3.57, 1H, br s, CH-12) and the 12-ketonic group was absent in **3**. Moreover, the substitution with -OH at position 12 can also be confirmed by HMBC (Figure 2) from H_3_-23 (δ_H_ 0.93, 3H, s) to C-12 (δ_C_ 72.0), as well the COSY correlation (Figure 2) H_2_-11/H-12. The configuration of **3** was confirmed to be unanimous as that of **2**. The NOESY correlations from H_3_-23 to H-12 supported an *S* assignment of oxymethine carbon at C-12, then established the structure illumination of lendenfeldarane G (Supplementary Materials, Figures S17–S24).

**Figure 5:**
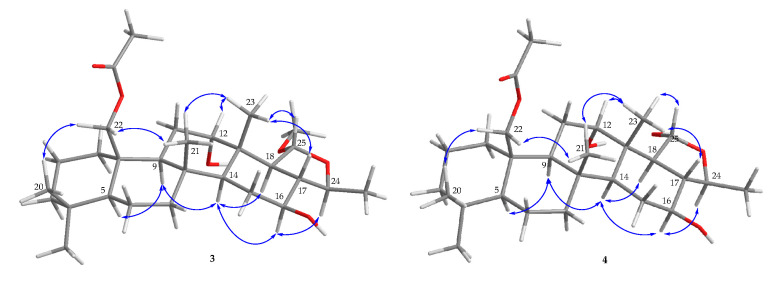
The selected NOESY correlations (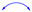) of **3** and **4**.

Lendenfeldarane H (**4**) was also obtained as an amorphous powder. The (+)-HRESIMS (*m/z* 501.31879, calculated for C_28_H_46_O_6_ + Na, 501.31866) and NMR data of **4** indicated a molecular formula C_28_H_46_O_6_ with six degrees of unsaturation. The IR spectra revealed the presence of OH (3292 cm^−1^) and ester carbonyl (1740 cm^−1^) groups. Based on the analysis of the NMR spectra between **3** and **4** (Table 2), a missing methoxy group signal was found, together with different assignments at position 25 (**4**: δ_H_ 5.35, 1H, dd, *J* = 6.8, 3.2 Hz/δ_C_ 96.5; **3**: δ_H_ 4.85, 1H, d, *J* = 6.4 Hz/δ_C_ 103.9). The HMBC cross peak (Figure 2) further revealed the replacement of an α-hydroxy group in **4**. The configuration of **4** was confirmed to be identical to that of **3** by NOESY experiment (Figure 5). Compound **4** was finally assigned, as shown in Figure 1 (Supplementary Materials, Figures S25–S32).

**Figure 6:**
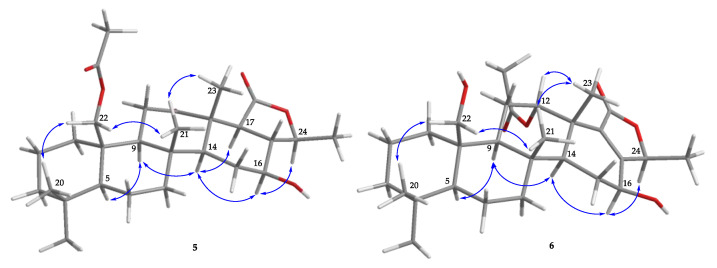
The selected NOESY correlations (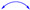) of **5** and **6**.

Lendenfeldarane I (**5**) was isolated as a white powder. The (+)-HRESIMS at *m*/*z* 497.28757 (calculated for C_28_H_42_O_6_ + Na, 497.28736) indicated a molecular formula of C_28_H_42_O_6_. The IR spectra showed absorptions for OH (3460 cm^−1^), C=O (1701 cm^−1^), and ester carbonyl (1745 cm^−1^) functionalities. The ^13^C NMR data (Table 3) revealed 28 carbons signals stored by HSQC and DEPT, including a ketone at δ_C_ 211.9 and two ester carbonyls at δ_C_ 170.7 and 172.3. Therefore, three degrees of unsaturation were built up, then the rest of the five unsaturated degrees were speculated to come from a pentacyclic homoscalarane. The 1D and 2D NMR data disclosed the existence of a compound **5**-like 6/6/6/6/5 pentacyclic skeleton. The only found divergence was located at E-ring, the disappearance of the ketal carbon in **2** was replaced by an ester carbonyl in **5**. Then the HMBC (Figure 2) cross-peak from H-24 to C-17, C-18, and C-25 allowed the establishment of a γ-valerolactone. The stereochemical configuration was identical to that of other scalarane sesterterpenes [5] based on the NOESY (Figure 6) correlations at H_3_-22/H_3_-20, H_2_-22/H_3_-21, H_3_-21/H_3_-23, H-5/H-9, H-9/H-14, H-14/16, H-14/H-18, and H-16/H-24. Consequently, compound **5** was assigned as shown in Figure 1 (Supplementary Materials, Figures S33–S40).

The molecular formula of lendenfeldarane J (**6**) was determined as C_28_H_42_O_6_ from an [M + Na]^+^ sodiated adduct ion at *m*/*z* 497.28729 (calcd. for C_28_H_42_O_6_ + Na, 497.28736) and NMR data, revealing eight degrees of unsaturation. The ^1^H NMR data (Table 3) of **6** showed the five uncoupled (singlet) methyls at δ_H_ 0.77, 0.87, 1.10, 1.16, and 1.98; one doublet coupled methyl at δ_H_ 1.41 (*J* = 6.6 Hz); and two oxymethines at δ_H_ 5.51(1H, t, *J* = 3.0 Hz) and 4.46 (1H, dd, *J* = 5.4, 5.4 Hz). The diastereotopic geminal proton at δ_H_ 3.88 (1H, dd, *J* = 11.4, 4.8 Hz) and 4.04 (1H, d, *J* = 11.4 Hz) were assumed to be an oxymethylene group. Based on the ^13^C spectrum, **6** was found to possess an oxymethylene (δ_C_ 62.8), two oxymethines (δ_C_ 61.7 and 75.5), two ester carbonyls (δ_C_ 170.5 and 169.8), as well as a tetra-substituted olefin (δ_C_ 135.7 and 161.3) that accounted for an unsaturated degree. Thus, the above NMR data and the remaining five unsaturated degrees of **6** required a pentacyclic analogue. Based on the COSY and HMBC correlations (Figure 2), the planer structure of **6** was determined as shown in Figure 1. Compound **6** held a methylfuran-2(*5H*)-one moiety determined by HMBC cross-correlations from H-24 to C-17, C-18, and C-25. Furthermore, the relative configuration was confirmed by NOESY (Figure 6) correlations (H-12/H_3_-23, H-16/H-14, and H-16/H-24) and the comparison with related compounds [5,21] (Supplementary Materials, Figures S41–S47).

Several lines of scientific and clinical evidences indicated that neutrophil oxidants and elastase secreted by inflammatory cells play critical roles in the pathogenesis of several inflammation-related disorders, such as psoriasis, arthritis, acute respiratory distress syndrome, and systemic lupus erythematos [22]. NADPH oxidase type 2 (NOX2) is an important enzyme that causes superoxide generation during respiratory burst, a predominant neutrophil function against foreign pathogens. An excessive amount of superoxide release can damage host tissues and lead to neutrophilic inflammation. Besides, another critical role, elastase, can contribute to neutrophil migration toward the inflammatory site, and activates neutrophil degranulation that causes the release of more elastolytic proteases to degrade the proteins from invading pathogens. Many recent studies have revealed that the pharmacological inhibition of NOX2 and elastase can restrict inflammatory responses, indicating the promising therapeutic potential of NOX2 and elastase inhibitors for treating neutrophil-dominant inflammatory disorders [23].

In the current study, the inhibition of fMLF-activated superoxide anion generation and elastase release were evaluated on metabolites **1**–**6** to characterize their property of anti-neutrophilic inflammation (Table 4). From these results, compound **1** showed the most potent inhibitory effect independently against elastase release, as well as a slight enhancing property in superoxide generation. With an additional acetyl functionality at C-22, compounds **2** and **3** both displayed activity of superoxide inhibition, but not elastase inhibition. These results suggest a crucial role of C-22-acetylation of homoscalarane on specifically affecting neutrophilic targets, such as NOX2 and elastase.

## 3. Material and Methods

### 3.1. General Experimental Procedures

Optical rotations spectra were recorded on a JASCO P-1010 polarimeter (cell length 10 mm) (JASCO, Tokyo, Japan). IR spectra were obtained with a Thermo Scientific Nicolet iS5 FT-IR spectrophotometer (Thermo Fisher Scientific, Waltham, MA, USA). ECD spectra were recorded on JASCO-815 CD spectrometer. The NMR spectra were obtained on a JEOL ECZ 400S or an ECZ 600R NMR (JEOL, Tokyo, Japan), using the residual CHCl_3_ signals (δ_H_ 7.26 ppm) and CDCl_3_ (δ_C_ 77.0 ppm) as the internal standards for ^1^H and ^13^C NMR, respectively. The coupling constants (*J*) are presented in Hz. ESIMS and HRESIMS data were collected on a Bruker 7 Tesla solariX FTMS system (Bruker, Bremen, Germany). TLC was performed on Kieselgel 60 F_254_ (0.25 mm, Merck, Darmstadt, Germany) and/or RP-18 F_254S_ (0.25 mm, Merck, Darmstadt, Germany) coated plates and then visualized by spraying with 10% H_2_SO_4_ and heating on a hot plate. Silica gel 60 (Merck, 40−63 and 63−200 μm) were used for column chromatography. Normal-phase HPLC (NP-HPLC) was performed using a system comprising a pump (L-7110; Hitachi, Tokyo, Japan), an injection port (Rheodyne, 7725; Rohnert Park, CA, USA), and a semi-preparative normal-phase column (YMC-Pack SIL, SIL-06, 250 × 20 mm, D. S-5 μm; Sigma-Aldrich, St. Louis, MO, USA). Reverse-phase HPLC (RP-HPLC) was performed using a system comprising a pump (L-2130; Hitachi), a photodiode array detector (L-2455; Hitachi), an injection port (Rheodyne; 7725), and a reverse-phase column (Luna 5 μm, C18(2) 100Å AXIA Packed, 250 × 21.2 mm; phenomenex, Torrance, CA, USA).

### 3.2. Animal Material

Specimen of the marine sponges *Lendenfeldia* sp. was collected by hand using self-contained underwater breathing apparatus (scuba) diving off the coast of Southern Taiwan on September 5, 2012, and stored in a freezer until extraction. The specimen was identified by one of the authors (Y.M. Huang). A voucher specimen (NMMBA-TWSP-12006) was deposited in the National Museum of Marine Biology and Aquarium, Pingtung, Taiwan.

### 3.3. Extraction and Isolation

Sliced bodies of *Lendenfeldia* sp. (wet weight 1.21 kg) were extracted with ethyl acetate (EtOAc). The EtOAc layer (5.09 g) was separated on silica gel and eluted using a mixture of hexanes and EtOAc (stepwise, 100:1—pure EtOAc) to yield 11 fractions A–K. Fraction I was separated by NP-HPLC using a mixture of dichloromethane and acetone (4:1, flow rate: 3.0 mL/min) to afford 22 fractions I1–I22. Fraction I4 was separated by RP-HPLC using a mixture of MeOH and H_2_O (85:15, flow rate: 5.0 mL/min) to afford **5** (1.3 mg). Fraction I6 was separated by RP-HPLC using a mixture of MeOH and H_2_O (8:2, flow rate: 5.0 mL/min) to afford **6** (0.2 mg). Fraction I17 was separated by RP-HPLC using a mixture of MeOH and H_2_O (8:2, flow rate: 5.0 mL/min) to afford **4** (3.2 mg). Fraction J was separated by NP-HPLC using a mixture of *n*-hexane and acetone (2:1, flow rate: 2.0 mL/min) to afford **1** (0.2 mg), **2** (10.0 mg) and **3** (14.8 mg).

Lendenfeldarane E (**1**): Amorphous powder; ${\left[\alpha \right]}_{D}^{25}$ −94 (*c* 0.07, CHCl_3_); IR (ATR) ν_max_ 3438, 1701 cm^−1^; ^1^H and ^13^C NMR spectroscopic data, see Table 1; ESIMS: *m/z* 455 [M + Na]^+^; HRESIMS: *m*/*z* 455.31340 (calcd. for C_27_H_44_O_4_ + Na, 455.31318).

Lendenfeldarane F (**2**): Amorphous powder; ${\left[\alpha \right]}_{D}^{25}$ +38 (*c* 0.50, CHCl_3_); IR (ATR) ν_max_ 3451, 1731, 1700 cm^−1^; ^1^H and ^13^C NMR spectroscopic data, see Table 1; ESIMS: *m/z* 513 [M + Na]^+^; HRESIMS: *m*/*z* 513.31878 (calcd. for C_29_H_46_O_6_ + Na, 513.31866).

Lendenfeldarane G (**3**): Amorphous powder; ${\left[\alpha \right]}_{D}^{25}$ +59 (*c* 0.74, CHCl_3_); IR (ATR) ν_max_ 3462, 1729 cm^−1^; ^1^H and ^13^C NMR spectroscopic data, see Table 2; ESIMS: *m/z* 515 [M + Na]^+^; HRESIMS: *m*/*z* 515.33437 (calcd. for C_29_H_48_O_6_ + Na, 515.33431).

Lendenfeldarane H (**4**): Amorphous powder; ${\left[\alpha \right]}_{D}^{25}$ +73 (*c* 0.16, CHCl_3_); IR (ATR) ν_max_ 3292, 1740 cm^−1^; ^1^H and ^13^C NMR spectroscopic data, see Table 2; ESIMS: *m/z* 501 [M + Na]^+^; HRESIMS: *m*/*z* 501.31879 (calcd. for C_28_H_46_O_6_ + Na, 501.31866).

Lendenfeldarane I (**5**): Amorphous powder; ${\left[\alpha \right]}_{D}^{25}$ +20 (*c* 0.07, CHCl_3_); IR (ATR) ν_max_ 3460, 1745, 1701 cm^−1^; ^1^H and ^13^C NMR spectroscopic data, see Table 3; ESIMS: *m/z* 497 [M + Na]^+^; HRESIMS: *m*/*z* 497.28757 (calcd. for C_28_H_42_O_6_ + Na, 497.28736).

Lendenfeldarane J (**6**): Amorphous powder; ${\left[\alpha \right]}_{D}^{25}$ −25 (c 0.07, CHCl_3_); IR (ATR) ν_max_ 3463, 1738 cm^−1^; ^1^H and ^13^C NMR spectroscopic data, see Table 3; ESIMS: *m/z* 497 [M + Na]^+^; HRESIMS: *m*/*z* 497.28729 (calcd. for C_28_H_42_O_6_ + Na, 497.28736).

### 3.4. ECD Calculations

The lowest energies of **1a** (5*S*,8*R*,9*S*,10*R*,13*S*,14*S*,16*S*,17*S*,18*S*,24*S*, and 25*R*) and **1b** (5*R*,8*S*,9*R*,10*S*, 13*R*,14*R*,16*R*,17*R*,18*R*,24*R*, and 25*S*) were calculated and the data were performed by the Gaussian 09 software (Gaussian Inc., Wallingford, CT, USA). The density functional theory (DFT) at the B3LYP/6-31G(d) level in the gas phase were used to obtain the restricted conformation. The final ECD files were generated by GaussSum 2.2.5 software with a bandwidth σ of 0.5 eV. The calculated ECD and experimental ECD curves were drawn by Excel.

### 3.5. Superoxide Anion Generation and Elastase Release by Human Neutrophils

Human neutrophils were obtained from healthy human volunteers and were isolated by Ficoll centrifugation and dextran sedimentation. Purified neutrophils were re-suspended in calcium (Ca^2+^)-free Hank’s balanced salt solution (HBSS) buffer at pH 7.4 and were maintained at 4 °C before use. For the superoxide anion generation assay, neutrophils (6 × 10^5^ cell/mL) were equilibrated in ferricytochrome *c* (0.6 mg/mL) and Ca^2+^ (1 mM) at 37 °C for 5 min and incubated with DMSO (0.1%) or tested compounds for another 5 min [24]. Cells were activated with formyl-methionyl-leucyl-phenylalanine (fMLF, 0.1 μM) for 10 min after priming with cytochalasin B (CB, 1 μg/mL) for 3 min. The change in absorbance was monitored continuously at 550 nm with a spectrophotometer (Hitachi U-3010). For the elastase release assay, neutrophils (6 × 10^5^ cell/mL) were equilibrated in MeO-Suc-Ala-Ala-Pro-Val-*p*-nitroanilide (100 μM) and Ca^2+^ (1 mM) at 37 °C for 5 min and incubated with dimethyl sulfoxide (DMSO) (0.1%) or test compounds for another 5 min. The cells were activated with fMLF (0.1 μM) for 10 min after the priming with CB (0.5 μg/mL) for 3 min. The change in absorbance was monitored continuously at 405 nm with a spectrophotometer [24]. The results are recorded as the mean ± SEM of three measurements. The inhibition % was measured at 10 μM concentration of each compound, and IC_50_ values were estimated from dose-response curves. Statistical analysis was carried out using Student’s *t*-tests with SigmaPlot (Systat Software, San Jose, CA, USA).

## 4. Conclusions

The current work is the first to illustrate the anti-neutrophilic inflammatory properties of scalarane-type sesterterpenoids, and reported a series of metabolites with novel structures, lendenfeldaranes E–J (**1**–**6**). These results also suggested a structural dependent specificity of C-22-acetylation in neutrophilic targets, which motivates future research to illustrate structural dependent specificity as well as further clarify the corresponding molecular mechanisms of the active leads.

## Figures and Tables

**Table 1:** ^1^H (400 MHz, CDCl_3_) and ^13^C (100 MHz, CDCl_3_) NMR data for 24-homoscalaranes **1** and **2**.

	1		2	
C/H	δ_H_ (*J* in Hz)	δ_C_ Mult.	δ_H_ (*J* in Hz)	δ_C_ Mult.
1	1.59 m; 0.80 m	39.4, CH_2_	1.99 m; 0.77 m	34.3, CH_2_
2	1.43 m; 1.58 m	18.1, CH_2_	1.46 m; 1.58 m	17.9, CH_2_
3	1.10 m; 1.36 m	41.8, CH_2_	1.14 m; 1.43 m	41.4, CH_2_
4		33.3, C		33.0, C
5	0.81 m	56.6, CH	0.97 m	56.9, CH
6	1.43 m	18.4, CH_2_	1.46 m	18.1, CH_2_
7	0.92 ddd (12.8, 12.8, 4.0)	41.9, CH_2_	0.98 m; 1.90 m	42.2, CH_2_
	1.82 ddd (12.8, 2.8, 2.8)			
8		37.8, C		37.7, C
9	1.15 m	61.3, CH	1.26 m	61.1, CH
10		38.1, C		41.2, C
11	2.66 dd (14.4, 13.6)	35.1, CH_2_	2.99 dd (14.8, 13.6)	37.9, CH_2_
	2.24 dd (13.6, 2.0)		2.43 dd (13.6, 2.4)	
12		215.3, C		214.8, C
13		51.8, C		51.9, C
14	1.13 m	59.6, CH	1.13 m	59.7, CH
15	1.52 m; 1.90 m	30.8, CH_2_	1.56 m; 1.92 m	31.0, CH_2_
16	3.43 ddd (10.4, 10.4, 4.4)	74.8, CH	3.43 ddd (10.0, 10.0, 4.4)	74.6, CH
17	1.89 m	49.6, CH	1.91 m	49.5, CH
18	1.62 m	53.0, CH	1.60 m	52.8, CH
19	0.82 s	21.3, CH_3_	0.84 s	21.8, CH_3_
20	0.85 s	33.2, CH_3_	0.87 s	33.7, CH_3_
21	1.07 s	17.1, CH_3_	1.15 s	16.5, CH_3_
22	0.86 s	15.7, CH_3_	4.19 dd (12.4, 1.6); 4.65 d (12.4)	64.6, CH_2_
23	1.36 s	15.3, CH_3_	1.37 s	15.4, CH_3_
24	3.84 qd (6.0, 2.4)	78.7, CH	3.83 qd (6.0, 2.0)	78.8, CH
25	5.35 d (4.0)	104.8, CH	5.33 d (4.4)	104.7, CH
26	1.40 d (6.0)	23.5, CH_3_	1.40 d (6.0)	23.5, CH_3_
22-OAc				170.7, C
			2.04 s	21.1, CH_3_
25-OMe	3.30 s	54.4, CH_3_	3.28 s	54.4, CH_3_

**Table 2:** ^1^H (400 MHz, CDCl_3_) and ^13^C (100 MHz, CDCl_3_) NMR data for 24-homoscalaranes **3** and **4**.

	3		4	
C/H	δ_H_ (*J* in Hz)	δ_C_ Mult.	δ_H_ (*J* in Hz)	δ_C_ Mult.
1	2.04 m; 0.82 m	34.5, CH_2_	2.03 m; 0.84 m	34.6, CH_2_
2	1.45 m; 1.56 m	18.4, CH_2_	1.45 m; 1.56 m	18.4, CH_2_
3	1.18 m; 1.43 m	41.5, CH_2_	1.17 br d (3.6); 1.42 m	41.5, CH_2_
4		32.9, C		33.0, C
5	1.10 m	56.6, CH	1.09 m	56.6, CH
6	1.56 m	18.1, CH_2_	1.56 m	18.1, CH_2_
7	1.09 m; 1.78 ddd (12.8, 3.2, 3.2)	42.0, CH_2_	1.08 m; 1.79 ddd (12.4, 3.2, 3.2)	42.1, CH_2_
8		37.6, C		37.6, C
9	1.57 m	52.0, CH	1.58 m	52.1, CH
10		40.1, C		40.2, C
11	1.89 m; 1.29 m	31.3, CH_2_	1.90 m; 1.29 m	31.3, CH_2_
12	3.57 br s	72.0, CH	3.67 ddd (3.2, 3.2, 3.2)	72.1, CH
13		39.0, C		39.0, C
14	1.38 m	52.2, CH	1.40 m	52.2, CH
15	1.86–1.98 m	25.9 CH_2_	1.91 m, 2.00 m	26.1 CH_2_
16	3.55 ddd (10.0, 10.0, 4.8)	72.9, CH	3.58 ddd (10.0, 10.0, 4.8)	73.0, CH
17	1.57 m	51.7, CH	1.58 m	52.1, CH
18	1.92 m	55.8, CH	1.94 m	56.7, CH
19	0.82 s	21.8, CH_3_	0.82 s	21.8, CH_3_
20	0.86 s	33.7, CH_3_	0.87 s	33.7, CH_3_
21	0.90 s	16.1, CH_3_	0.91 s	16.1, CH_3_
22	4.17 d (11.6); 4.56 d (11.6)	65.0, CH_2_	4.18 dd (12.0, 0.8); 4.57 d (12.0)	65.0, CH_2_
23	0.93 s	16.3, CH_3_	0.94 s	16.2, CH_3_
24	3.98 qd (6.0, 3.2)	79.5, CH	4.09 qd (6.0, 3.2)	79.7, CH
25	4.85 d (6.4)	103.9, CH	5.35 dd (6.8, 3.2)	96.5, CH
26	1.37 d (6.0)	20.3, CH_3_	1.36 d (6.0)	20.5, CH_3_
22-OAc		171.0, C		171.0, C
	2.05 s	21.2, CH_3_	2.06 s	21.2, CH_3_
25-OMe	3.45 s	56.6, CH_3_		

**Table 3:** ^1^H and ^13^C NMR data for 24-homoscalaranes **5** and **6**.

	5		6	
C/H	δ_H_ (*J* in Hz) ^a^	δ_C_ Mult. ^b^	δ_H_ (*J* in Hz) ^c^	δ_C_ Mult. ^d^
1	1.99 m; 0.80 m	34.4, CH_2_	2.10 m; 0.51 ddd (12.6, 12.6, 3.6)	34.3, CH_2_
2	1.49 m; 1.64 m	18.0, CH_2_	1.43 m; 1.56 m	18.1, CH_2_
3	1.15 m; 1.45 m	41.4, CH_2_	1.13 m; 1.44 m	41.7, CH_2_
4		33.0, C		33.0, C
5	0.97 m	57.0, CH	0.98 dd (12.6, 2.4)	57.0, CH
6	1.50 m; 1.60 m	18.1, CH_2_	1.42 m; 1.57 m	17.9, CH_2_
7	0.99 m; 1.87 m	41.9, CH_2_	1.12 m, 1.90 m	42.0, CH_2_
8		38.5, C		37.0, C
9	1.28 br d (14.0)	63.3, CH	1.31 dd (7.2, 7.2)	53.5, CH
10		41.6, C		41.8, C
11	3.15 dd (14.0, 12.4); 2.51 dd (12.4, 2.4)	37.6, CH_2_	1.87–1.95 m	27.9, CH_2_
12		211.9, C	5.51 t (3.0)	73.8, CH
13		50.0, C		39.0, C
14	0.95 m	59.3, CH	1.79 dd (12.6, 2.4)	46.2, CH
15	1.50 m; 1.97 m	31.1, CH_2_	2.21 m	23.9 CH_2_
16	3.61 ddd (9.6, 9.6, 4.4)	72.0, CH	4.46 dd (5.4, 5.4)	61.7, CH
17	1.92 m	51.4, CH		161.3, C
18	2.58 d (14.4)	49.7, CH		135.7, C
19	0.84 s	21.8, CH_3_	0.77 s	21.8, CH_3_
20	0.87 s	33.7, CH_3_	0.87 s	33.8, CH_3_
21	1.18 s	16.6, CH_3_	1.10 s	16.3, CH_3_
22	4.17 dd (11.6, 1.6); 4.67 d (11.6)	64.6, CH_2_	3.88 dd (11.4, 4.8); 4.04 d (11.4)	62.8, CH_2_
23	1.38 s	14.7, CH_3_	1.16 s	19.4, CH_3_
24	4.30 qd (6.0, 2.4)	79.5, CH	5.07 q (6.6)	76.5, CH
25		172.3, C		170.5, C
26	1.53 d (6.0)	20.1, CH_3_	1.41 d (6.6)	18.3, CH_3_
12-OAc				169.8, C
			1.98 s	21.2, CH_3_
22-OAc		170.7, C		
	2.06 s	21.1, CH_3_		

^a^ 400 MHz, CDCl_3_. ^b^ 100 MHz, CDCl_3_. ^c^ 600 MHz, CDCl_3_. ^d^ 150 MHz, CDCl_3_.

**Table 4:** Inhibitory effects of 24-homoscalaranes **1**–**6** on superoxide anion generation and elastase release by human neutrophils in response to fMLF.

Compound	Superoxide Anions			Elastase Release		
	IC_50_ (μM) ^a^	Inh (Enh) ^b^ %		IC_50_ (μM) ^a^	Inh %	
1		(11.35 ± 3.65)	*	1.74 ± 0.08	82.80 ± 3.91	***
2	6.17 ± 0.16	70.68 ± 3.86	***		26.15 ± 3.40	**
3	6.81 ± 0.52	62.92 ± 2.58	***		25.19 ± 4.01	**
4		7.13 ± 3.69			2.97 ± 1.63	
5		9.97 ± 4.38			6.81 ± 2.46	
6		4.52 ± 2.91			1.16 ± 0.89	

Percentage of inhibition (Inh %) at 10 μM. Results are presented as mean ± S.E.M. (*n* = 3~5). * *p* < 0.05, ** *p* < 0.01, *** *p* < 0.001 compared with the control (DMSO). ^a^ Concentration necessary for 50 % inhibition (IC_50_). ^b^ Inh = inhibition, Enh = Enhancement.

## Supplementary Materials

Supplementary Materials according to this article can be found online at https://www.mdpi.com/1660-3397/18/9/434/s1. HRESIMS, 1D and 2D NMR spectra of compounds **1**–**6**.
