# Supplementary material for: Sponge-Derived 24-Homoscalaranes as Potent Anti-Inflammatory Agents
Source: Mar Drugs. 2020 Aug 19;18(9):434. doi: 10.3390/md18090434 (PMC7551342; doi:10.3390/md18090434)
Supplement: Supplementary file 1 [file marinedrugs-18-00434-s001.pdf]

Supporting Information

No	Content	page
S1.	HRESIMS spectrum of compound **1**.	3
S2.	^{1}H NMR spectrum (400 MHz) of compound **1** in $CDCl_3$.	3
S3.	^{13}C NMR spectrum (100 MHz) of compound **1** in $CDCl_3$.	4
S4.	DEPT spectrum (100 MHz) of compound **1** in $CDCl_3$.	4
S5.	HSQC spectrum of compound **1** in $CDCl_3$.	5
S6.	HMBC spectrum of compound **1** in $CDCl_3$.	5
S7.	^{1}H–^{1}H COSY spectrum of compound **1** in $CDCl_3$.	6
S8.	NOESY spectrum of compound **1** in $CDCl_3$.	6
S9.	HRESIMS spectrum of compound **2**.	7
S10.	^{1}H NMR spectrum (400 MHz) of compound **2** in $CDCl_3$.	7
S11.	^{13}C NMR spectrum (100 MHz) of compound **2** in $CDCl_3$.	8
S12.	DEPT spectrum (100 MHz) of compound **2** in $CDCl_3$.	8
S13.	HSQC spectrum of compound **2** in $CDCl_3$.	9
S14.	HMBC spectrum of compound **2** in $CDCl_3$.	9
S15.	^{1}H–^{1}H COSY spectrum of compound **2** in $CDCl_3$.	10
S16.	NOESY spectrum of compound **2** in $CDCl_3$.	10
S17.	HRESIMS spectrum of compound **3**.	11
S18.	^{1}H NMR spectrum (400 MHz) of compound **3** in $CDCl_3$.	11
S19.	^{13}C NMR spectrum (100 MHz) of compound **3** in $CDCl_3$.	12
S20.	DEPT spectrum (100 MHz) of compound **3** in $CDCl_3$.	12
S21.	HSQC spectrum of compound **3** in $CDCl_3$.	13
S22.	HMBC spectrum of compound **3** in $CDCl_3$.	13
S23.	^{1}H–^{1}H COSY spectrum of compound **3** in $CDCl_3$.	14
S24.	NOESY spectrum of compound **3** in $CDCl_3$.	14
S25.	HRESIMS spectrum of compound **4**.	15
S26.	^{1}H NMR spectrum (400 MHz) of compound **4** in $CDCl_3$.	15
S27.	^{13}C NMR spectrum (100 MHz) of compound **4** in $CDCl_3$.	16
S28.	DEPT spectrum (100 MHz) of compound **4** in $CDCl_3$.	16
S29.	HSQC spectrum of compound **4** in $CDCl_3$.	17
S30.	HMBC spectrum of compound **4** in $CDCl_3$.	17
S31.	^{1}H–^{1}H COSY spectrum of compound **4** in $CDCl_3$.	18
S32.	NOESY spectrum of compound **4** in $CDCl_3$.	18
S33	HRESIMS spectrum of compound **5**.	19
S34	^{1}H NMR spectrum (400 MHz) of compound **5** in $CDCl_3$.	19
S35	^{13}C NMR spectrum (100 MHz) of compound **5** in $CDCl_3$.	20
S36	DEPT spectrum (100 MHz) of compound **5** in $CDCl_3$.	20
S37	HSQC spectrum of compound **5** in $CDCl_3$.	21
S38	HMBC spectrum of compound **5** in $CDCl_3$.	21
S39	^{1}H–^{1}H COSY spectrum of compound **5** in $CDCl_3$.	22

S40	NOESY spectrum of compound **5** in $CDCl_3$.	22
S41	HRESIMS spectrum of compound **6**.	23
S42	^{1}H NMR spectrum (600 MHz) of compound **6** in $CDCl_3$.	23
S43	^{13}C NMR spectrum (150 MHz) of compound **6** in $CDCl_3$.	24
S44	HSQC spectrum of compound **6** in $CDCl_3$.	24
S45	HMBC spectrum of compound **6** in $CDCl_3$.	25
S46	^{1}H–^{1}H COSY spectrum of compound **6** in $CDCl_3$.	25
S47	NOESY spectrum of compound **6** in $CDCl_3$.	26

Mass Spectrum SmartFormula Report

Analysis Info

Analysis Name	D:\Data\a2\R101021_000005.d	11/25/2019 12:59:34 PM
Method	broadband first signal	Operator: YU HSIAO-CHING
Sample Name	R-10-10-2-1	Instrument: BRUKER FT-MS solariX
Comment	ESI Positive	

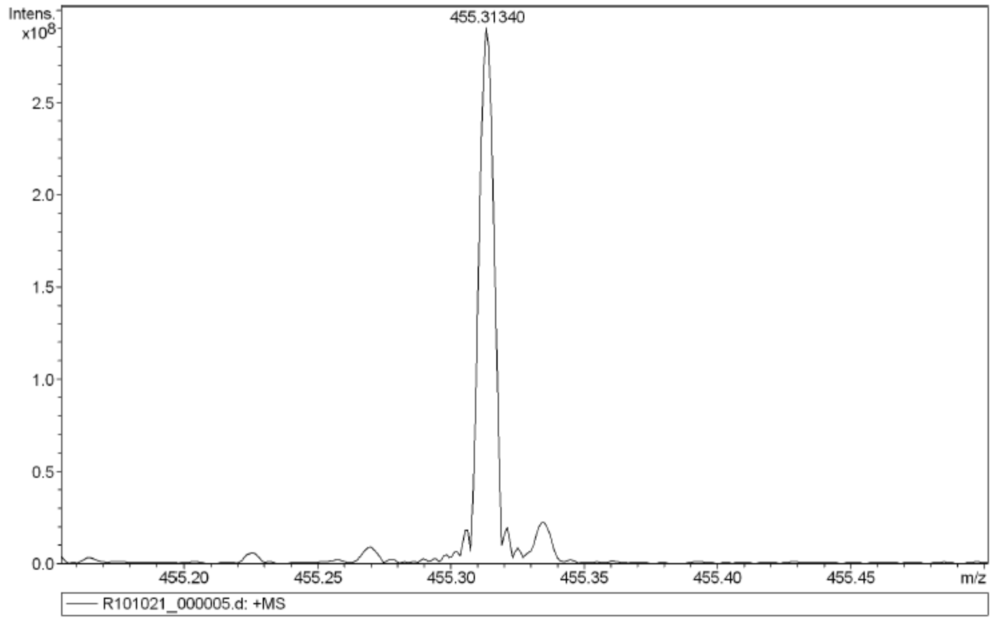

Meas. m/z	#	Formula	Score	m/z	err [mDa]	err [ppm]	mSigma	rdb	e⁻ Conf	N-Rule
455.31340	1	C 27 H 44 Na O 4	100.00	455.31318	-0.21	-0.47	10.0	5.5	even	ok

S1. HRESIMS spectrum of compound **1**.

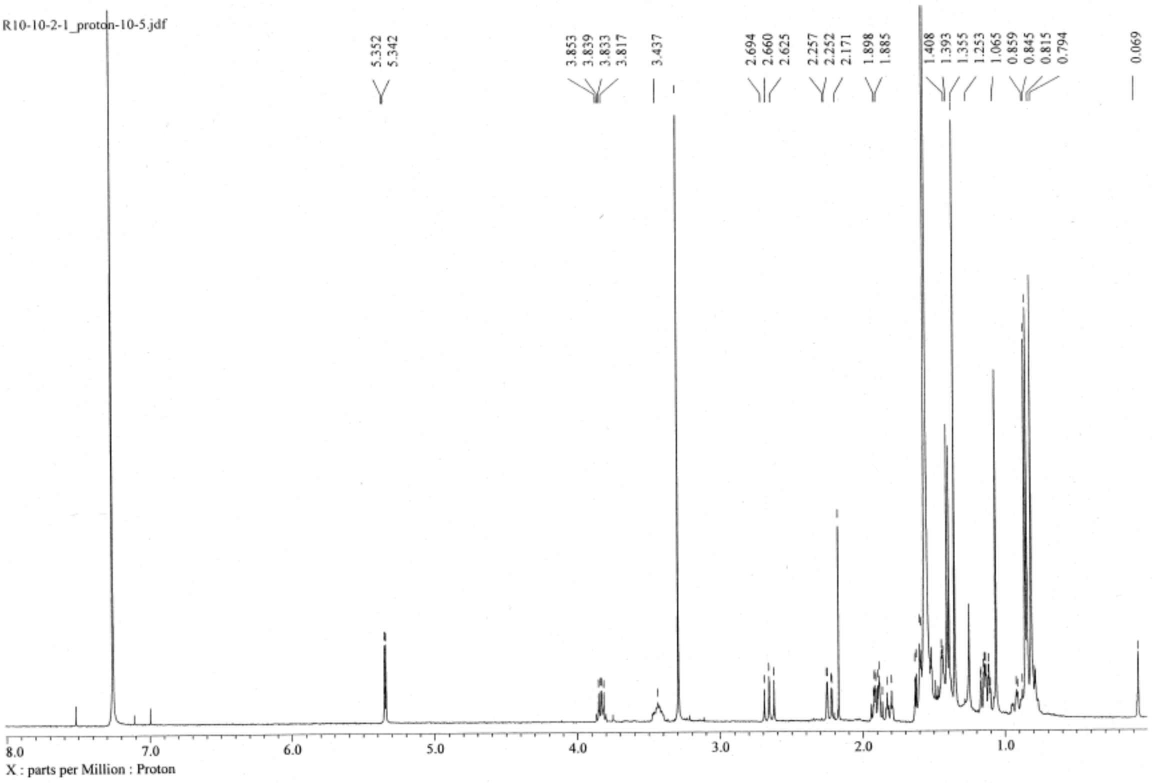

S2. ^{1}H NMR spectrum (400 MHz) of compound **1** in $CDCl_3$.

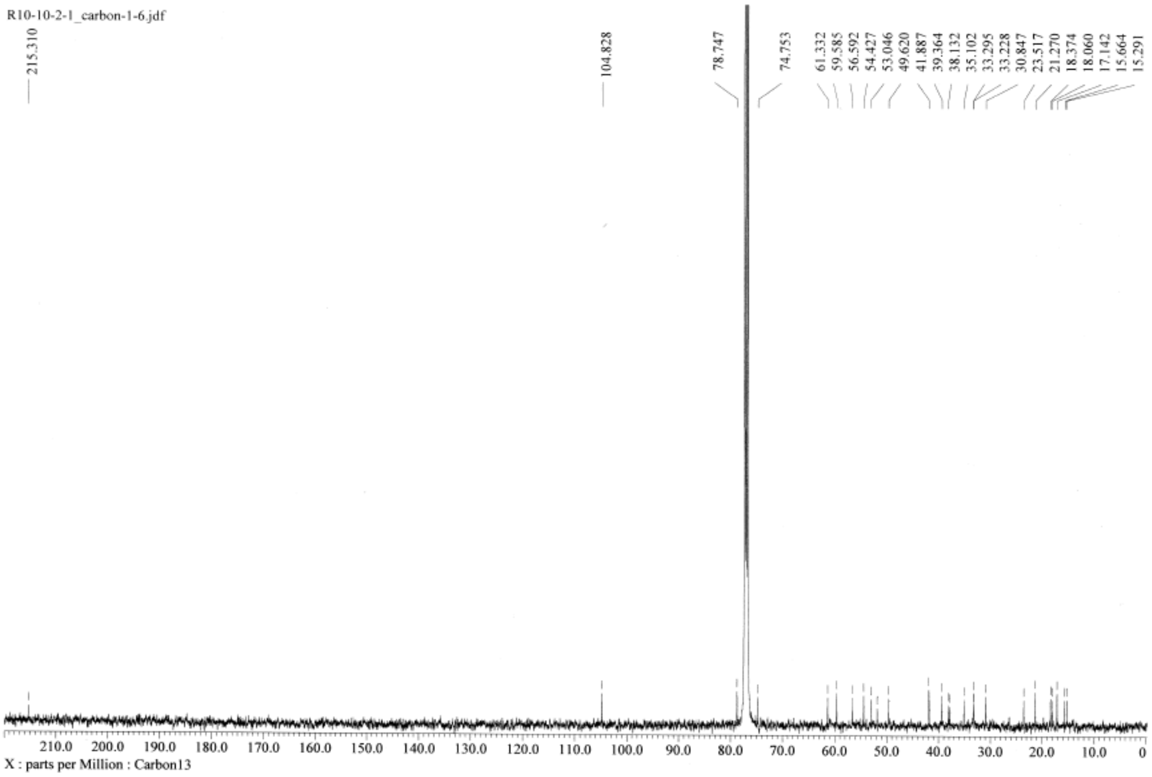

S3. ^{13}C NMR spectrum (100 MHz) of compound **1** in $CDCl_3$.

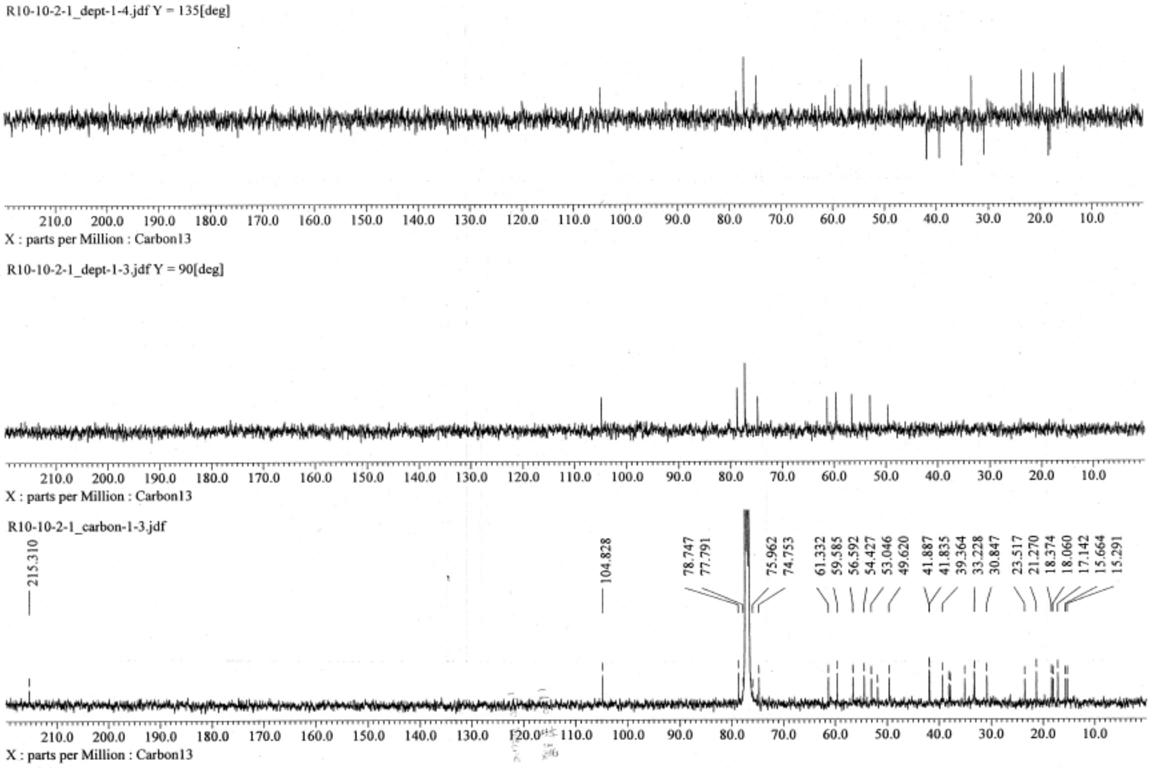

S4. DEPT spectrum (100 MHz) of compound **1** in CDC_3

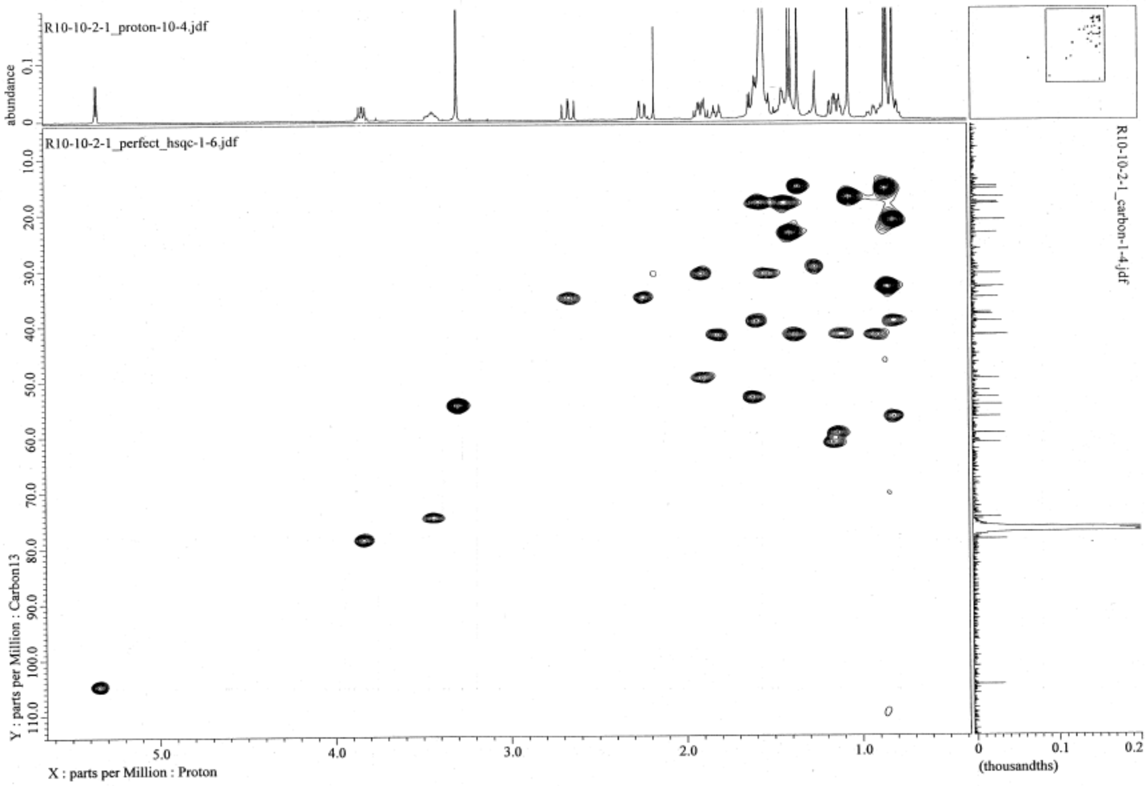

S5. HSQC spectrum of compound **1** in $CDCl_3$.

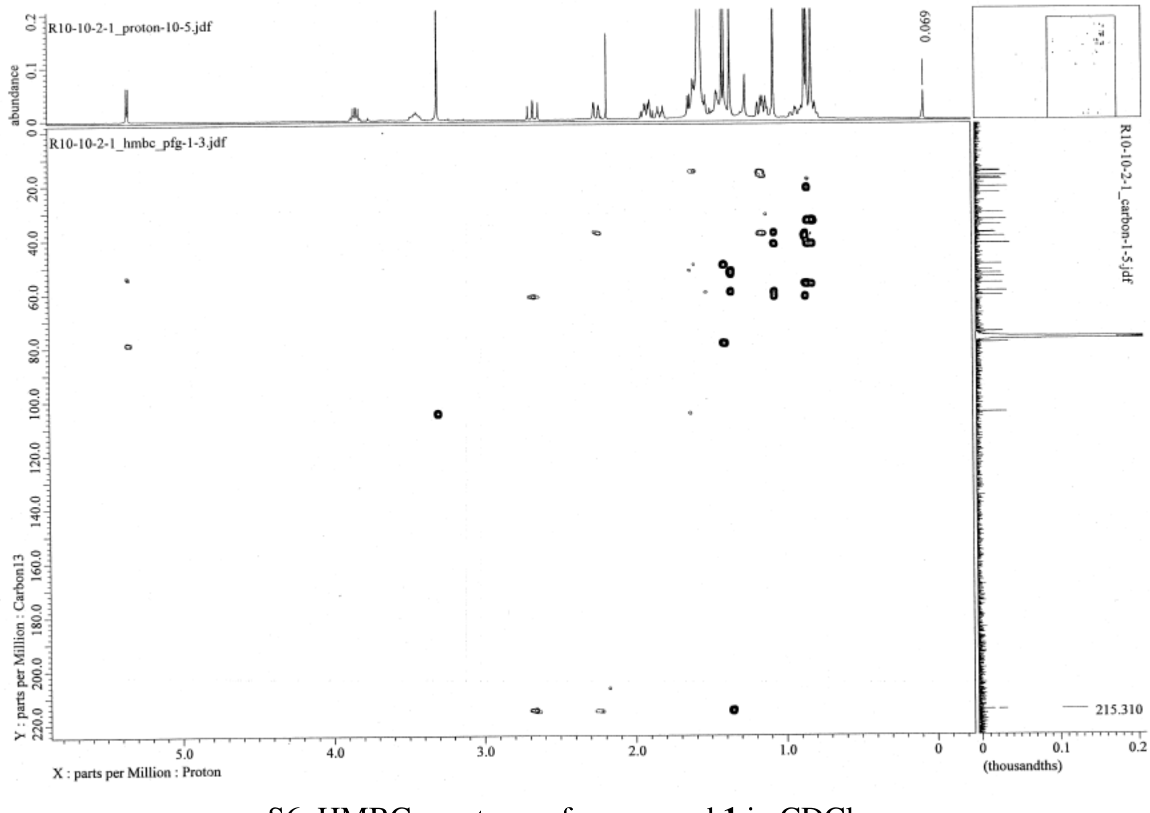

S6. HMBC spectrum of compound **1** in $CDCl_3$.

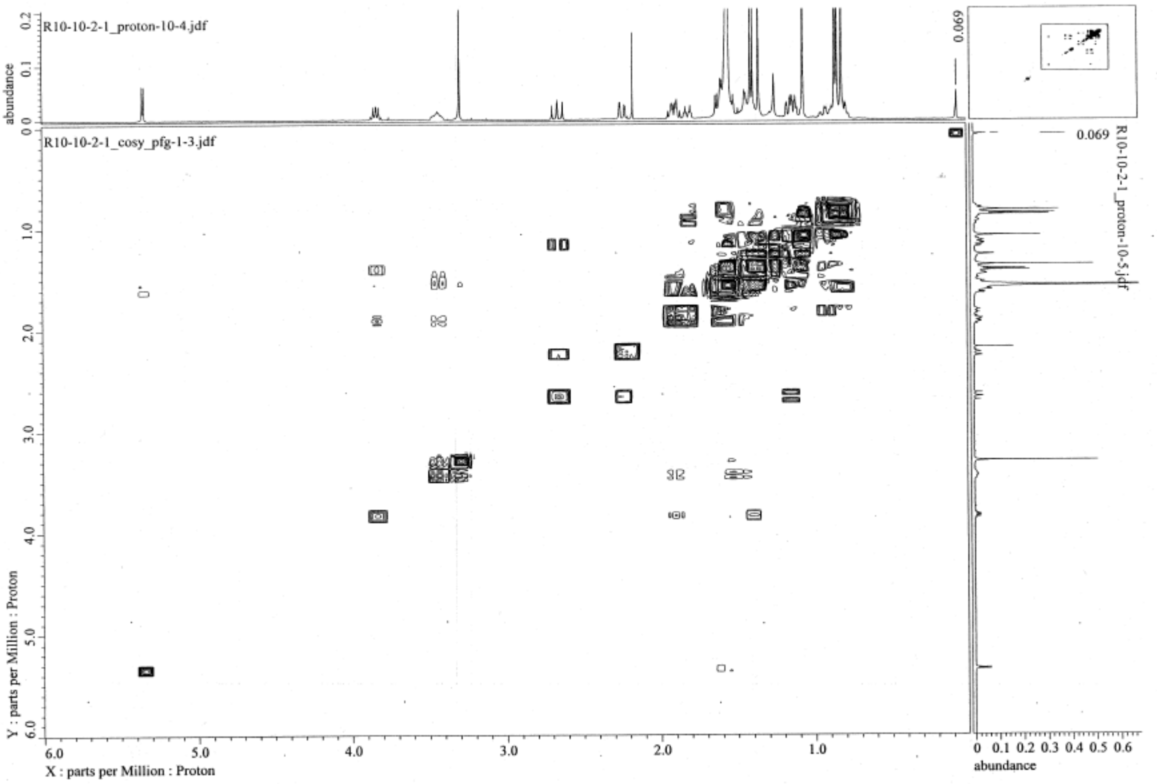

S7. ^1H-^1H COSY spectrum of compound **1** in $CDCl_3$.

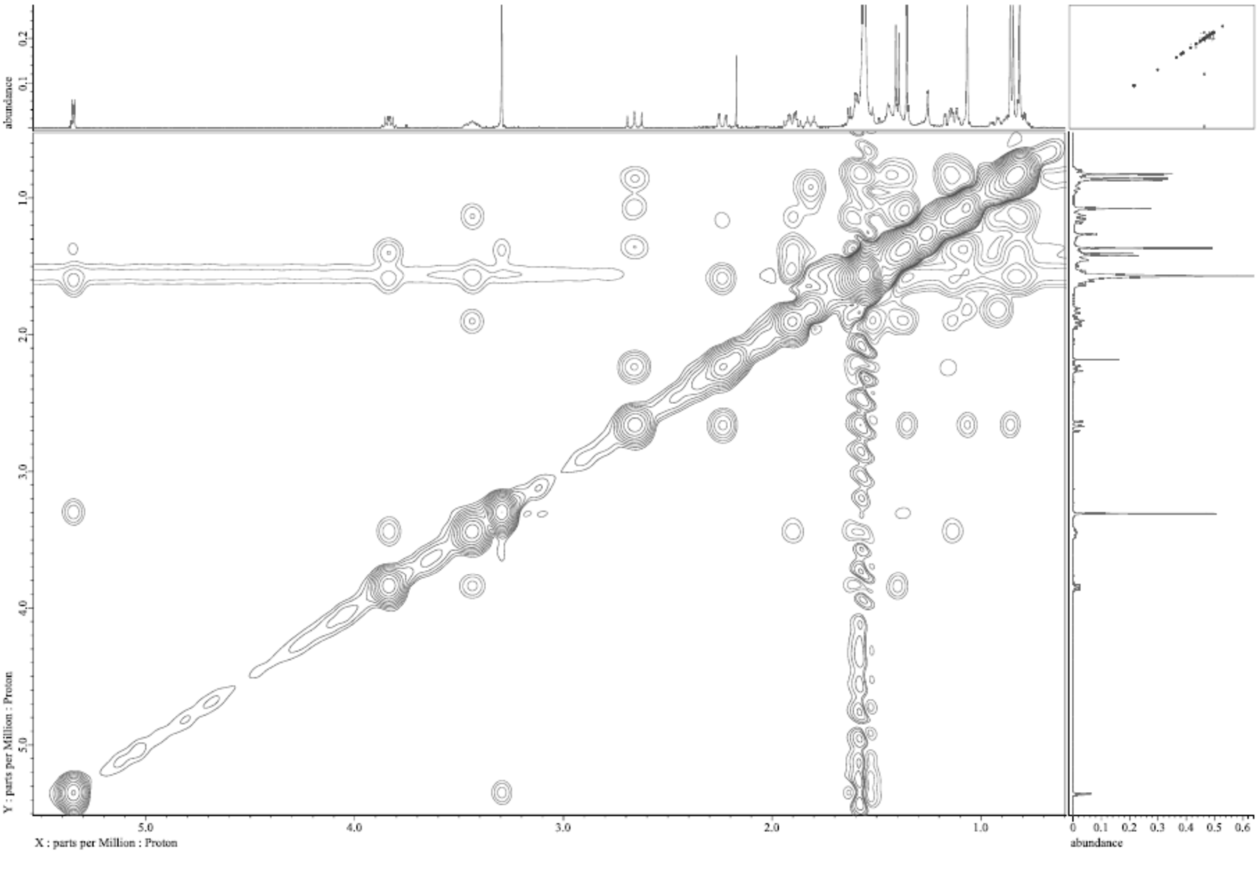

S8. NOESY spectrum of compound **1** in $CDCl_3$

Mass Spectrum SmartFormula Report

Analysis Info

Analysis Name	D:\Data\a2\R10104_000002.d	11/25/2019 1:08:58 PM
Method	broadband first signal	Operator: YU HSIAO-CHING
Sample Name	R-10-10-4	Instrument: BRUKER FT-MS solariX
Comment	ESI Positive	

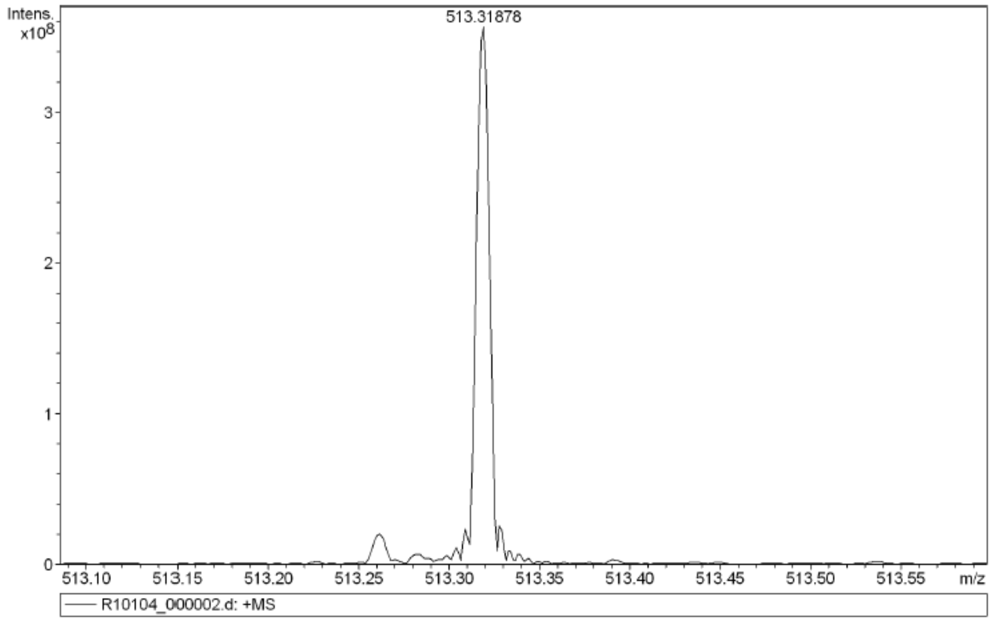

Meas. m/z	#	Formula	Score	m/z	err [mDa]	err [ppm]	mSigma	rdb	e⁻ Conf	N-Rule
513.31878	1	C 29 H 46 Na O 6	100.00	513.31866	-0.12	-0.23	7.4	6.5	even	ok

S9. HRESIMS spectrum of compound **2**.

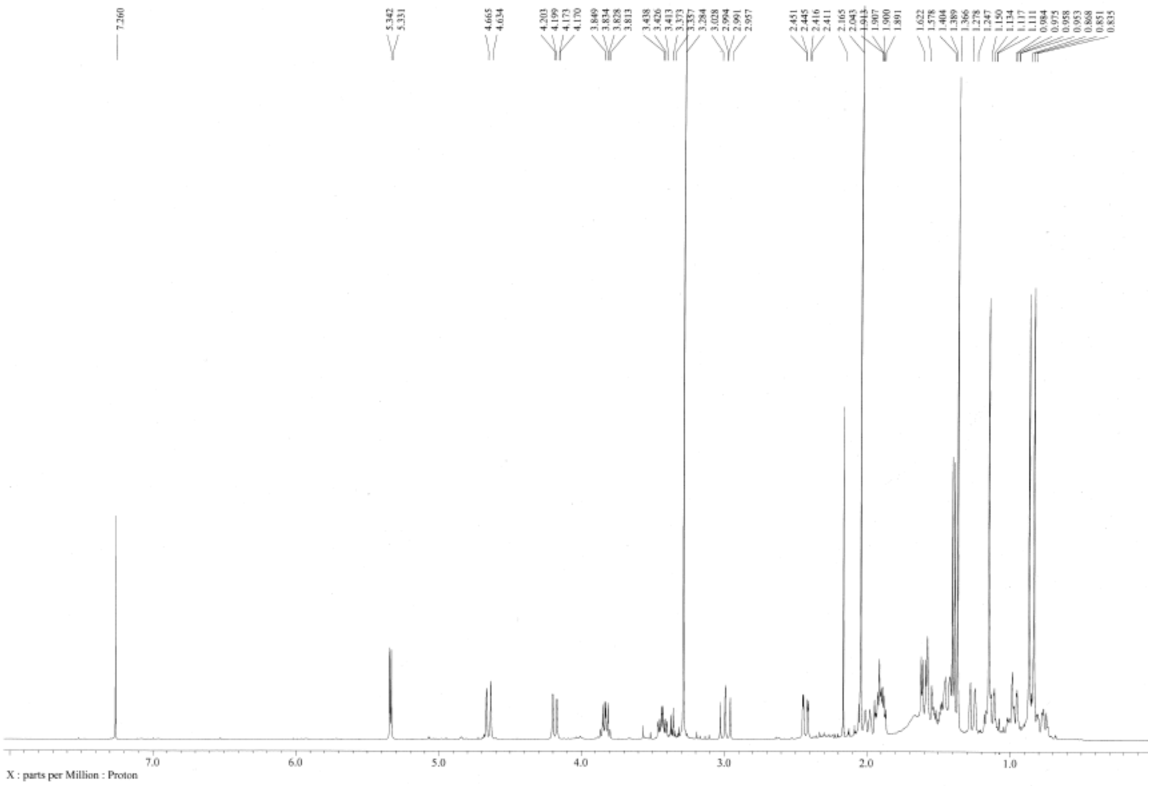

S10. ^{1}H NMR spectrum (400 MHz) of compound **2** in $CDCl_3$.

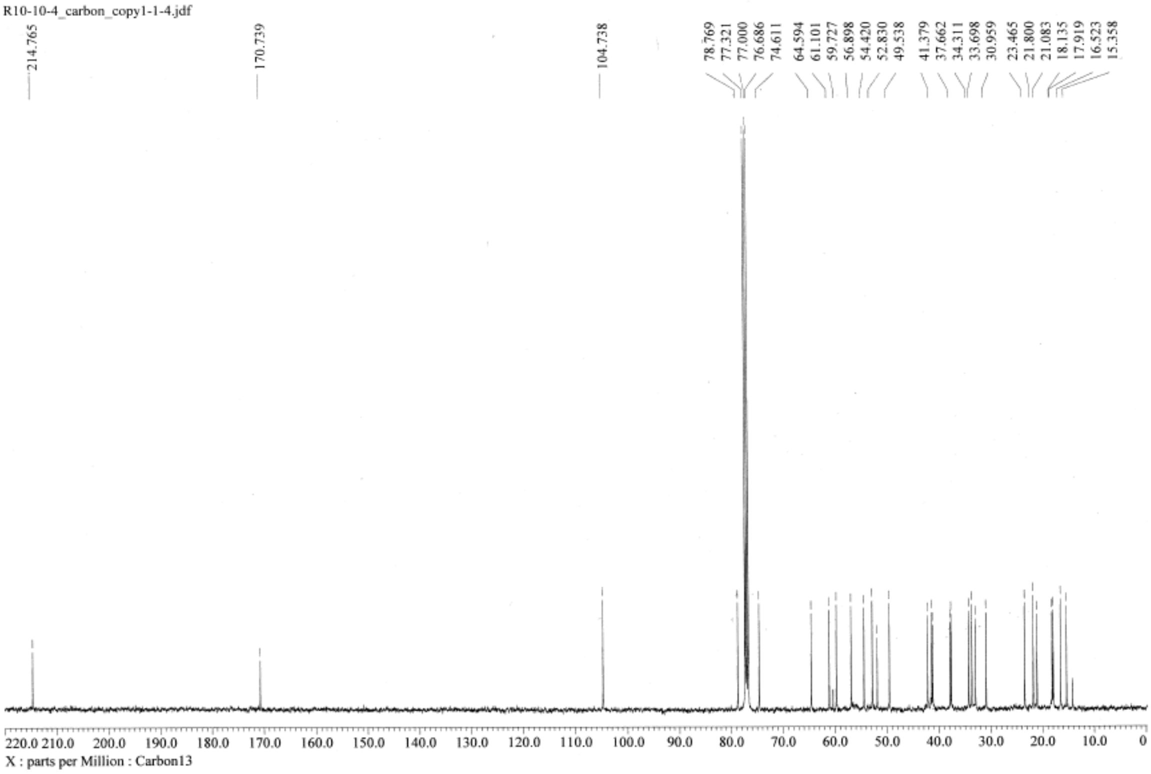

S11. ^{13}C NMR spectrum (100 MHz) of compound **2** in $CDCl_3$.

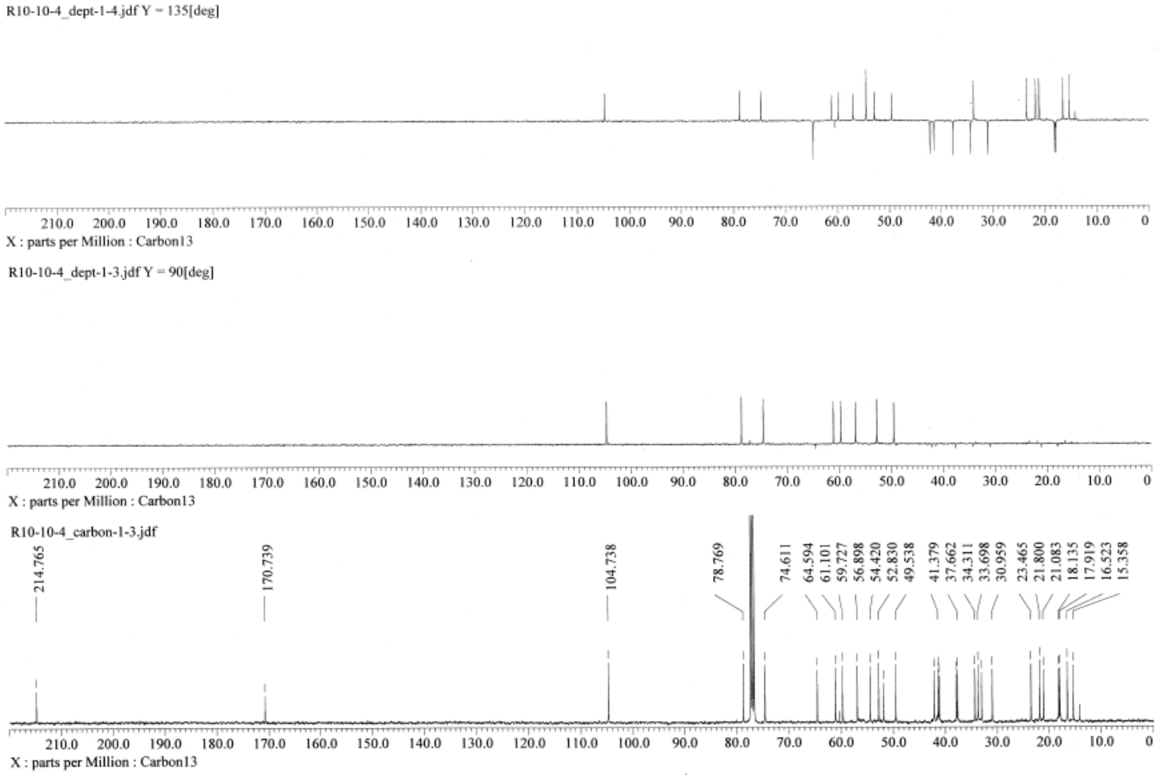

S12. DEPT spectrum (100 MHz) of compound **2** in $CDCl_3$.

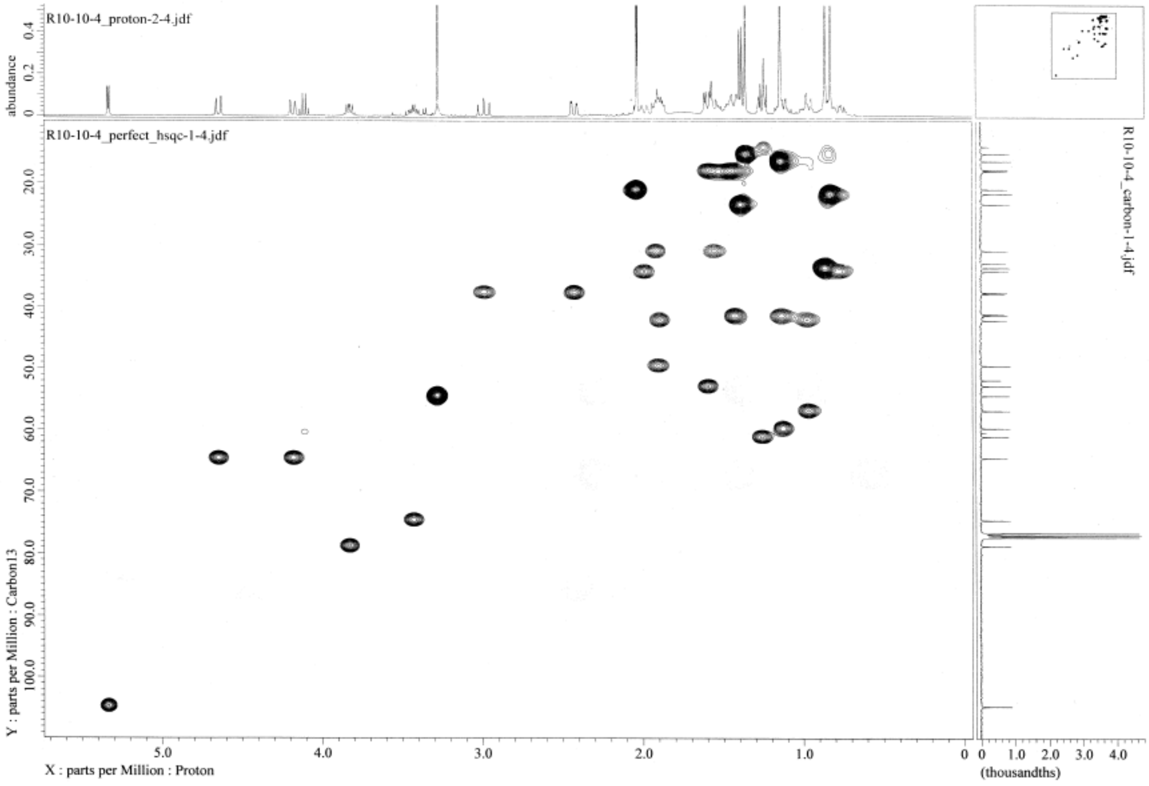

S13. HSQC spectrum of compound **2** in $CDCl_3$.

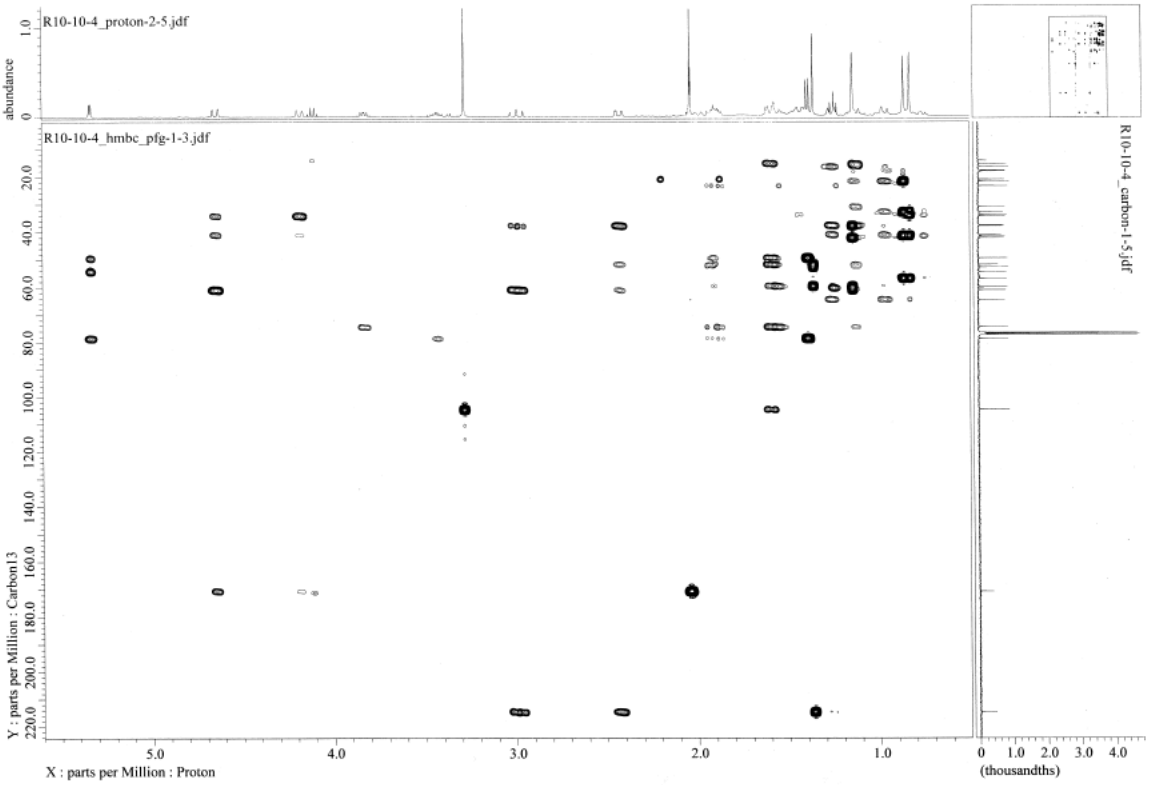

S14. HMBC spectrum of compound **2** in $CDCl_3$.

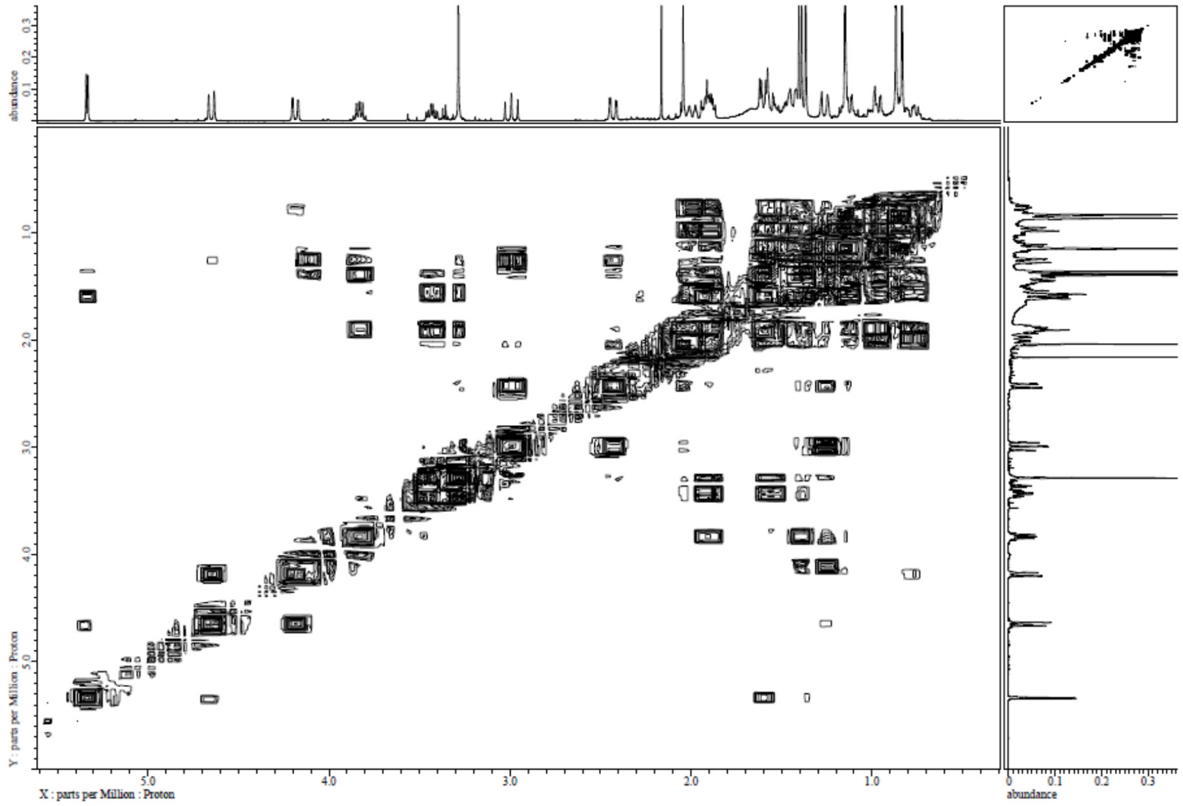

S15. ^{1}H-^{1}H COSY spectrum of compound **2** in $CDCl_3$.

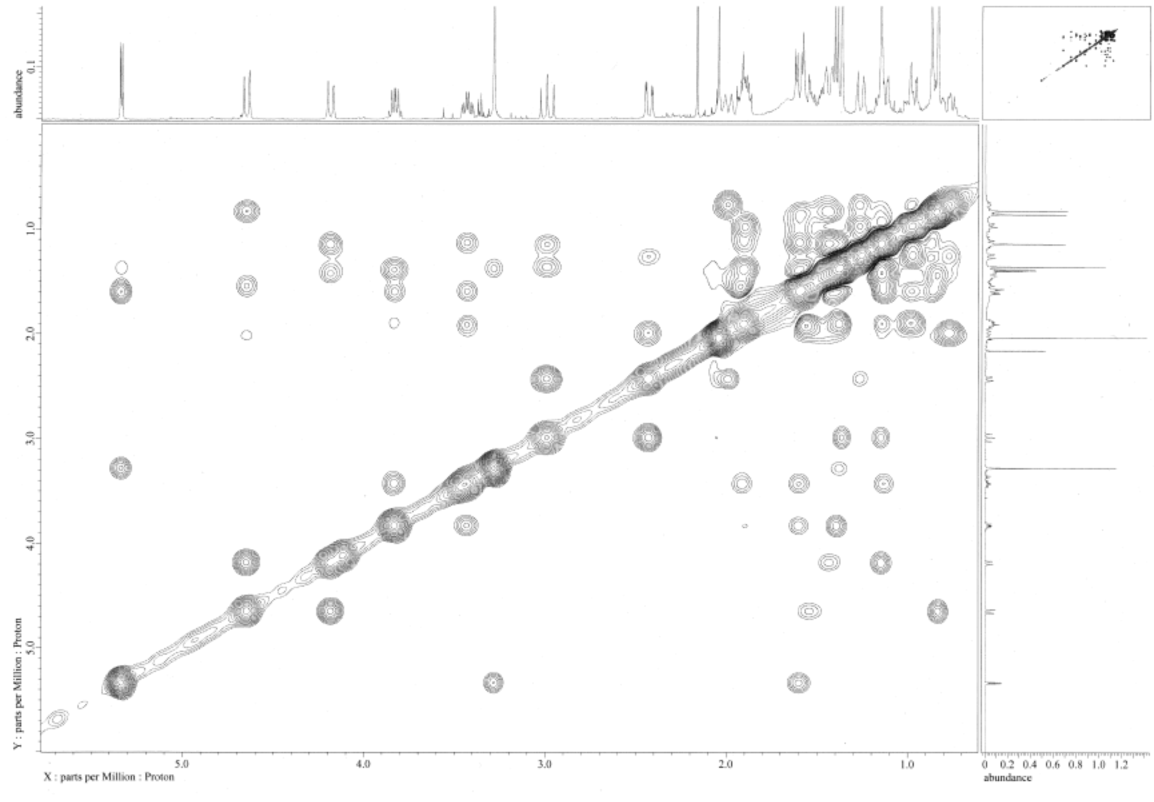

S16 NOESY spectrum of compound **2** in $CDCl_3$

Mass Spectrum SmartFormula Report

Analysis Info

Analysis Name	D:\Data\a2\R10103_000002.d	11/25/2019 1:03:34 PM
Method	broadband first signal	Operator: YU HSIAO-CHING
Sample Name	R-10-10-3	Instrument: BRUKER FT-MS solariX
Comment	ESI Positive	

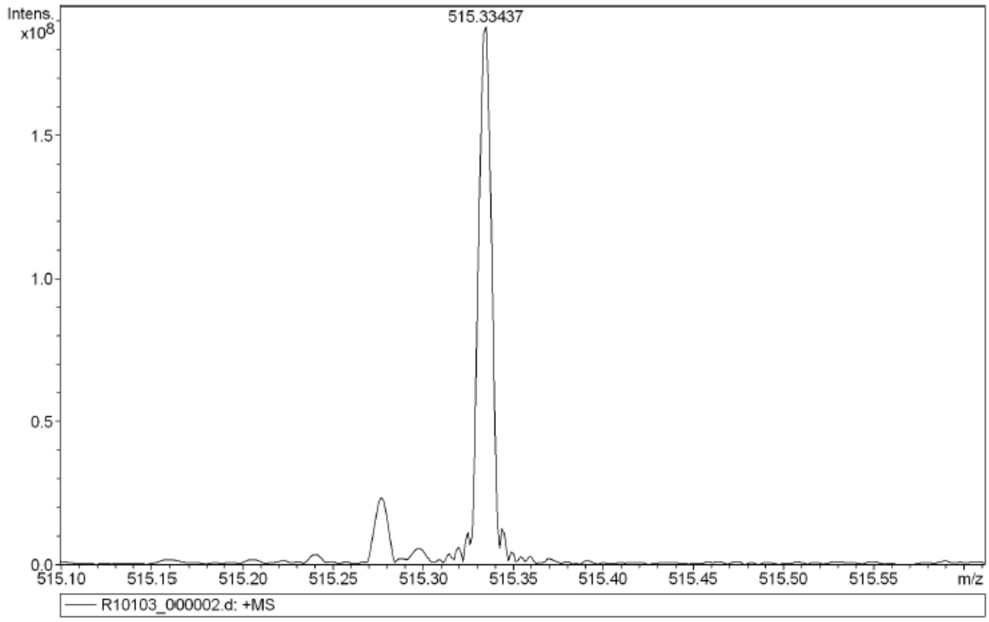

Meas. m/z	#	Formula	Score	m/z	err [mDa]	err [ppm]	mSigma	rdb	e⁻ Conf	N-Rule
515.33437	1	C 29 H 48 Na O 6	100.00	515.33431	-0.06	-0.12	10.5	5.5	even	ok

S17. HRESIMS spectrum of compound **3**.

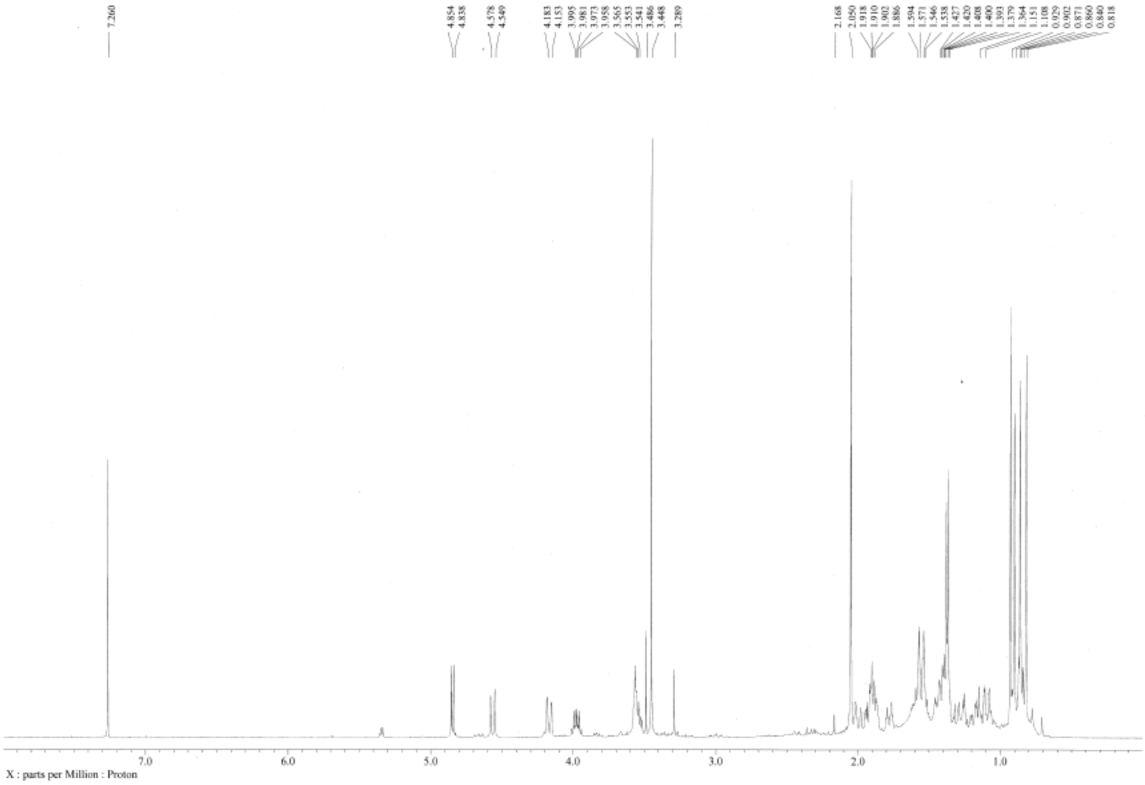

S18. ^{1}H NMR spectrum (400 MHz) of compound **3** in $CDCl_3$.

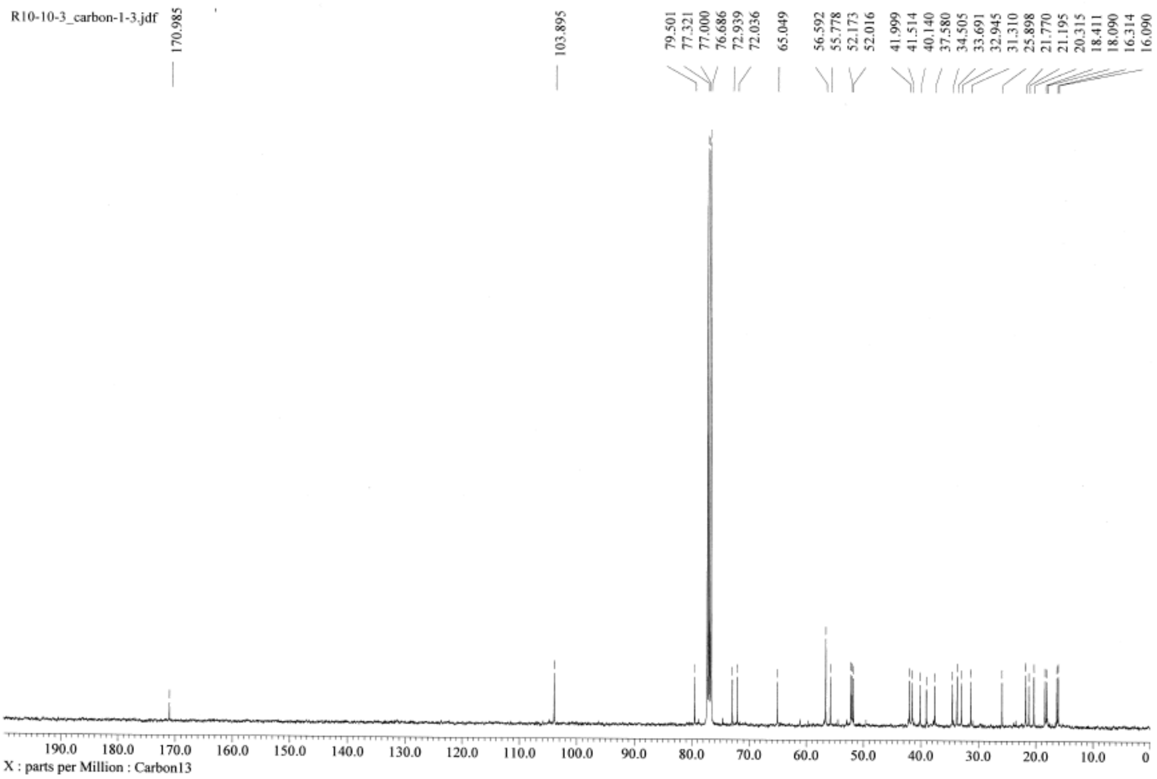

S19. ^{13}C NMR spectrum (125 MHz) of compound **3** in $CDCl_3$.

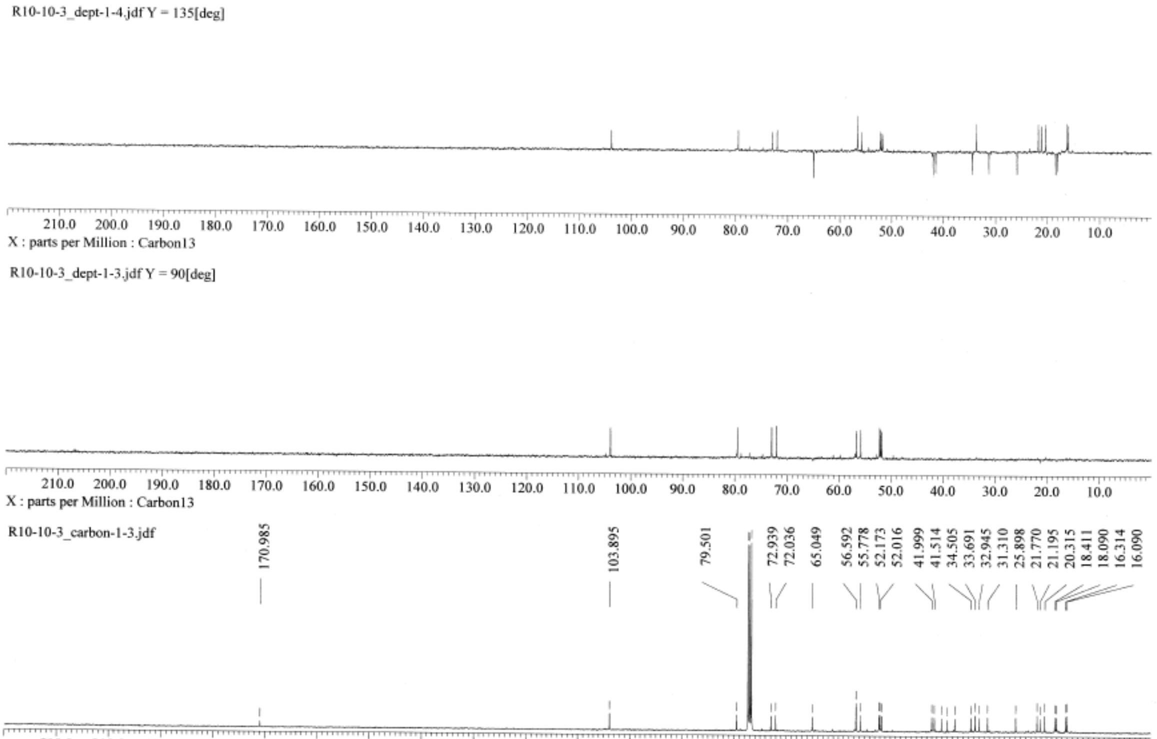

S20. DEPT spectrum (100 MHz) of compound **3** in $CDCl_3$.

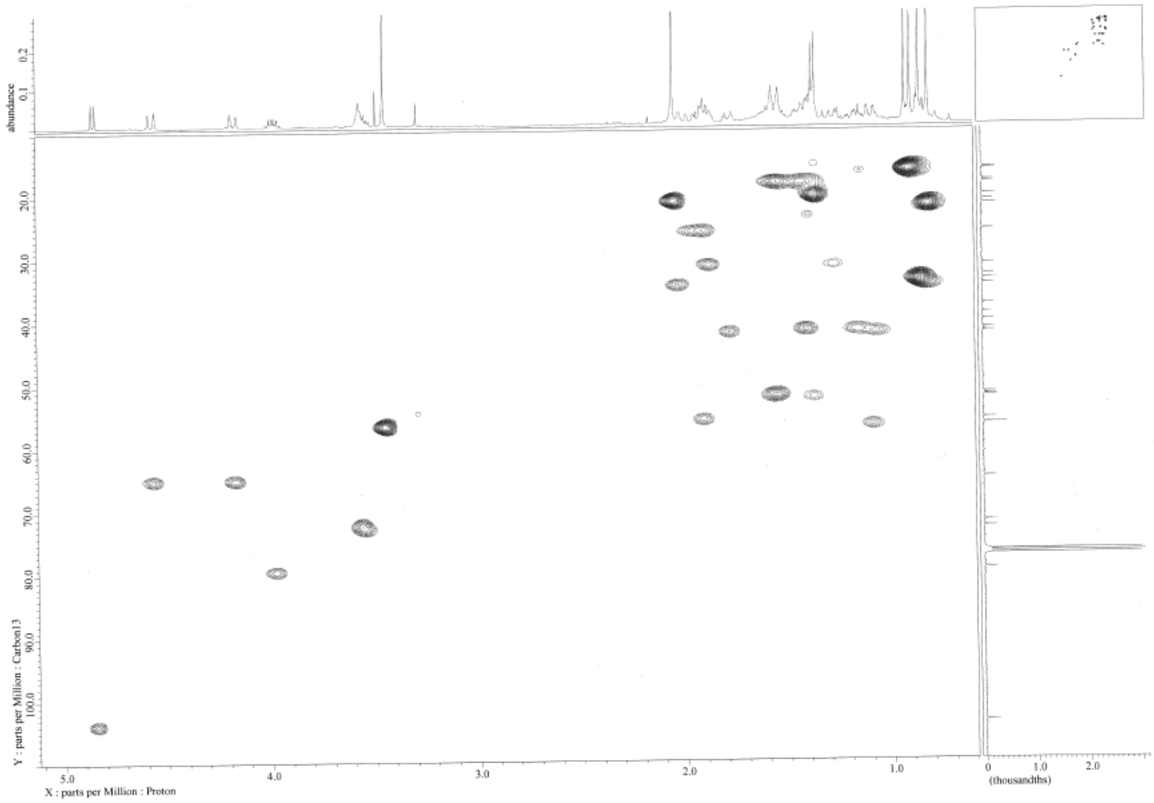

S21. HSQC spectrum of compound **3** in $CDCl_3$.

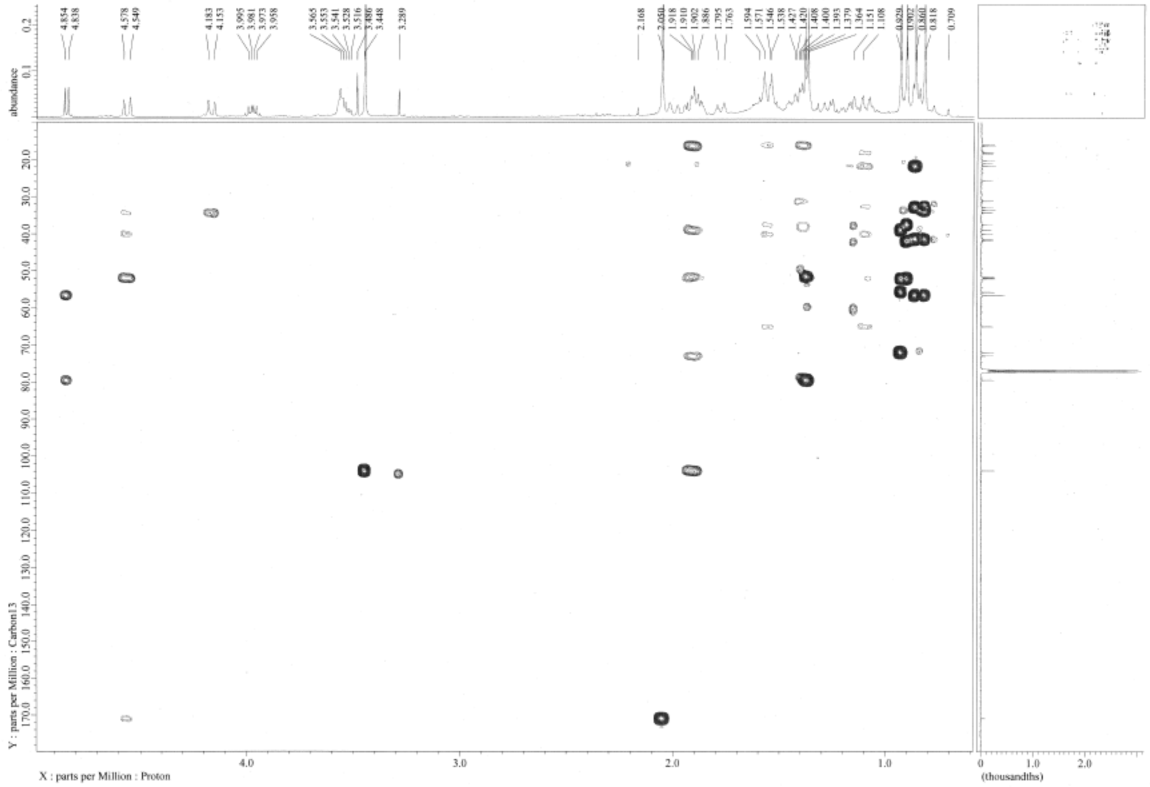

S22. HMBC spectrum of compound **3** in $CDCl_3$.

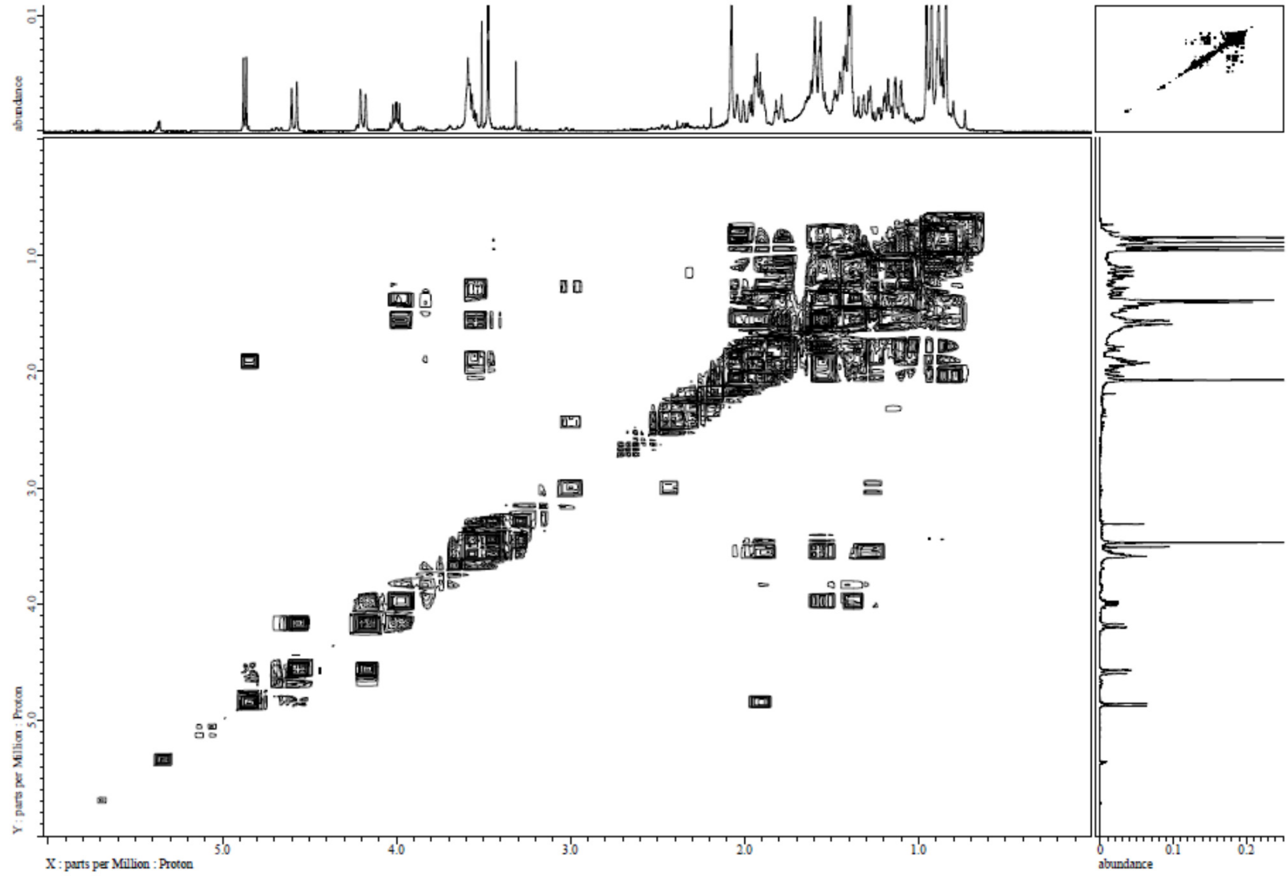

S23. ^{1}H-^{1}H COSY spectrum of compound **3** in $CDCl_3$.

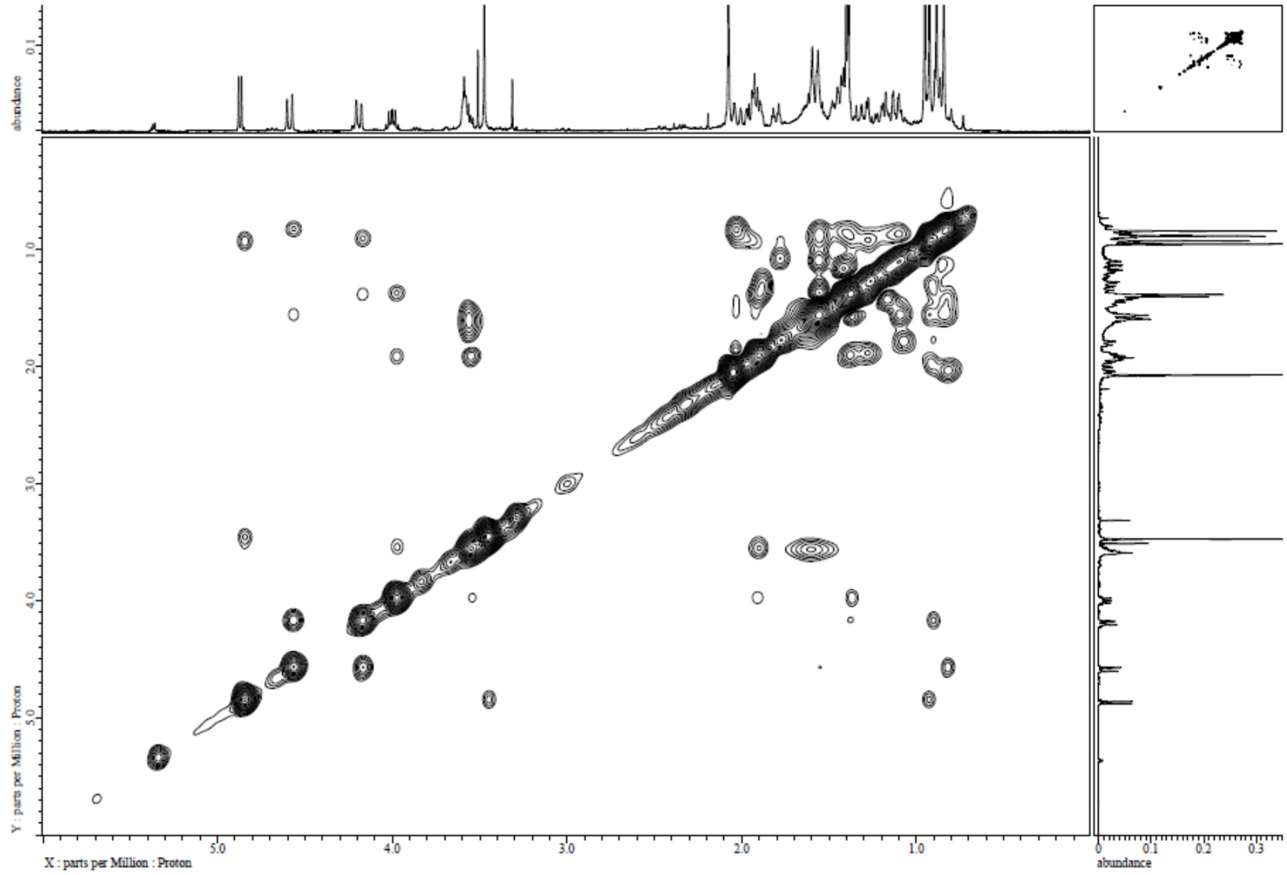

S24. NOESY spectrum of compound **3** in $CDCl_3$

Mass Spectrum SmartFormula Report

Analysis Info

Analysis Name	D:\Data\a2\R9172_000002.d	12/10/2019 4:47:05 PM
Method	broadband first signal	Operator: YU HSIAO-CHING
Sample Name	R9-17-2	Instrument: BRUKER FT-MS solariX
Comment	ESI Positive	

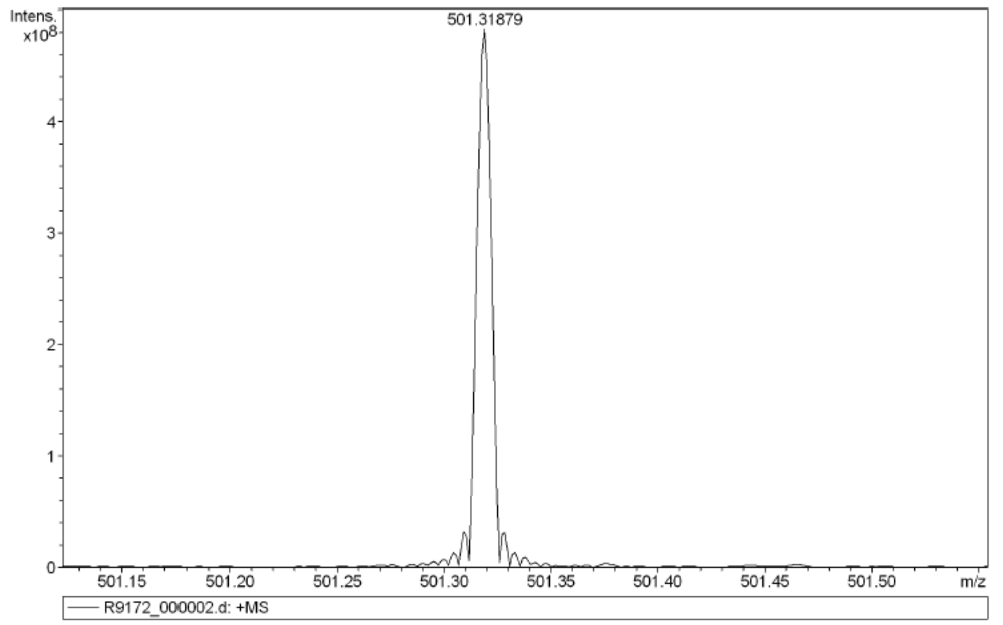

Meas. m/z	#	Formula	Score	m/z	err [mDa]	err [ppm]	mSigma	rdb	e⁻ Conf	N-Rule
501.31879	1	C 28 H 46 Na O 6	100.00	501.31866	-0.13	-0.26	10.5	5.5	even	ok

S25. HRESIMS spectrum of compound **4**.

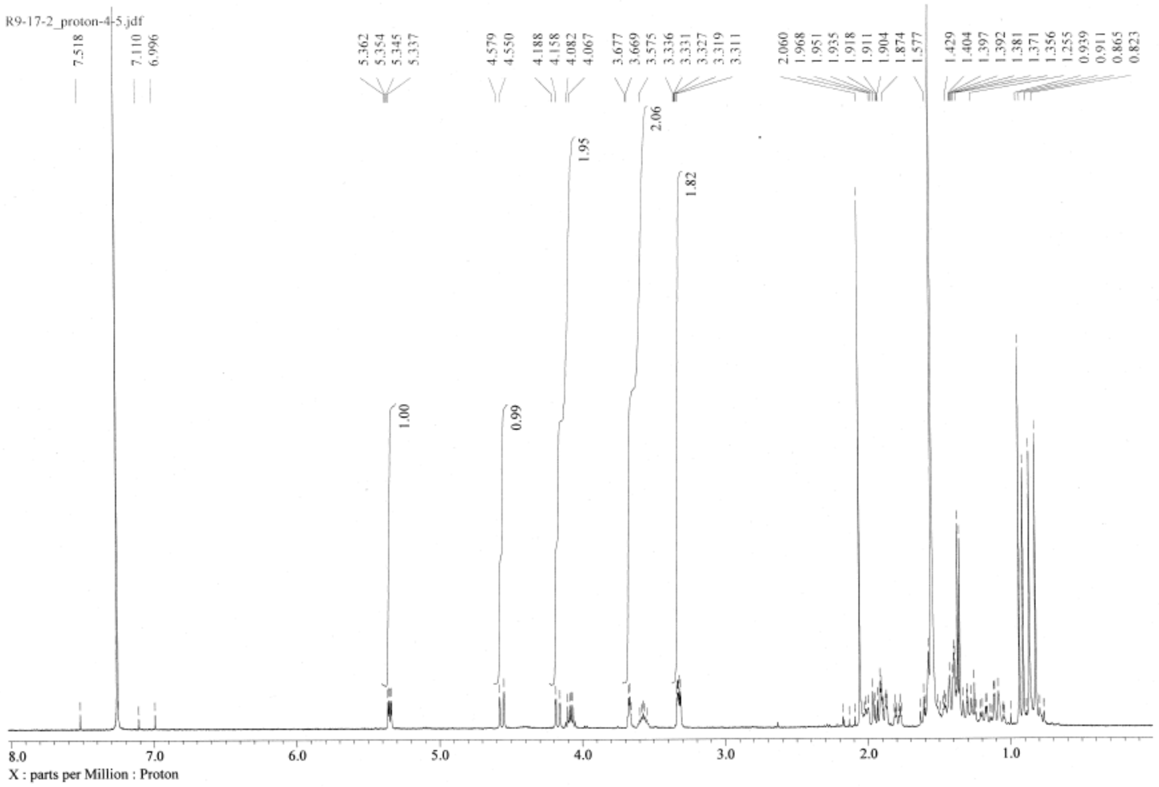

S26. ^{1}H NMR spectrum (400 MHz) of compound **4** in $CDCl_3$.

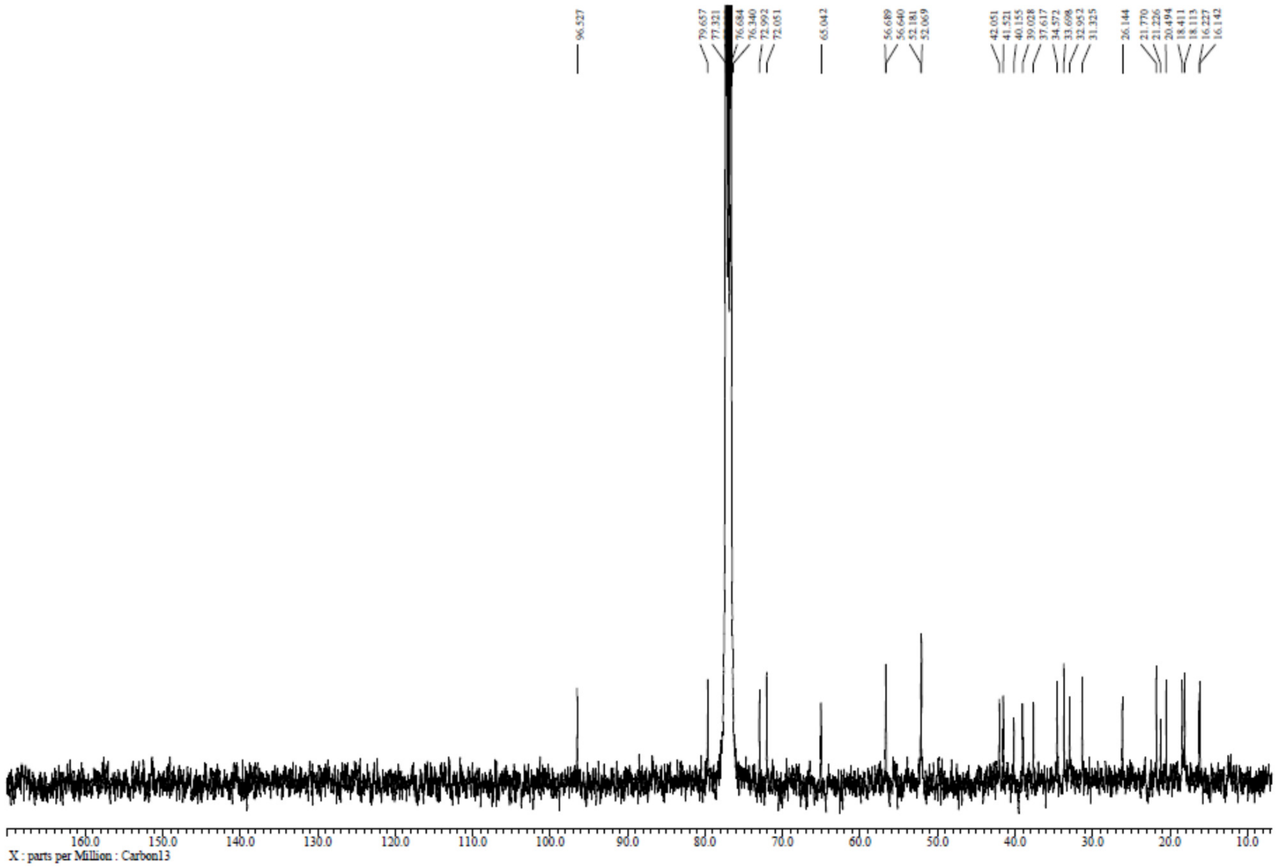

S27. ^{13}C NMR spectrum (100 MHz) of compound **4** in $CDCl_3$.

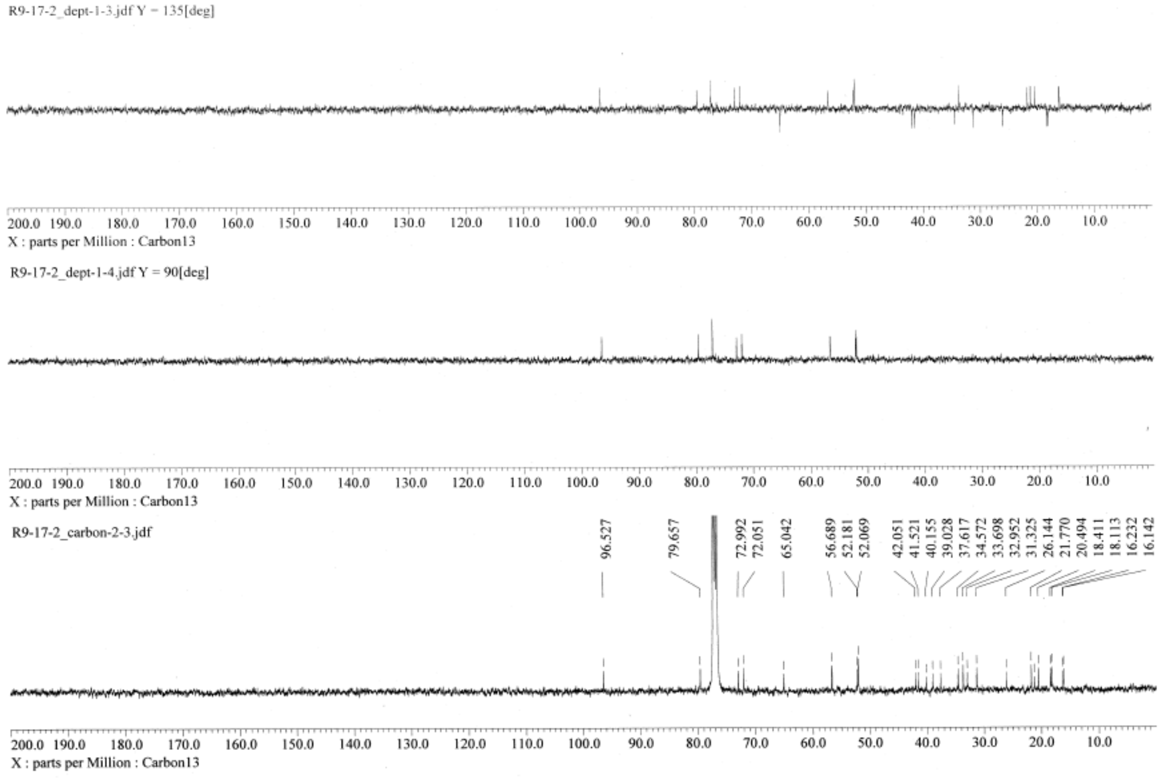

S28. DEPT spectrum (100 MHz) of compound **4** in $CDCl_3$.

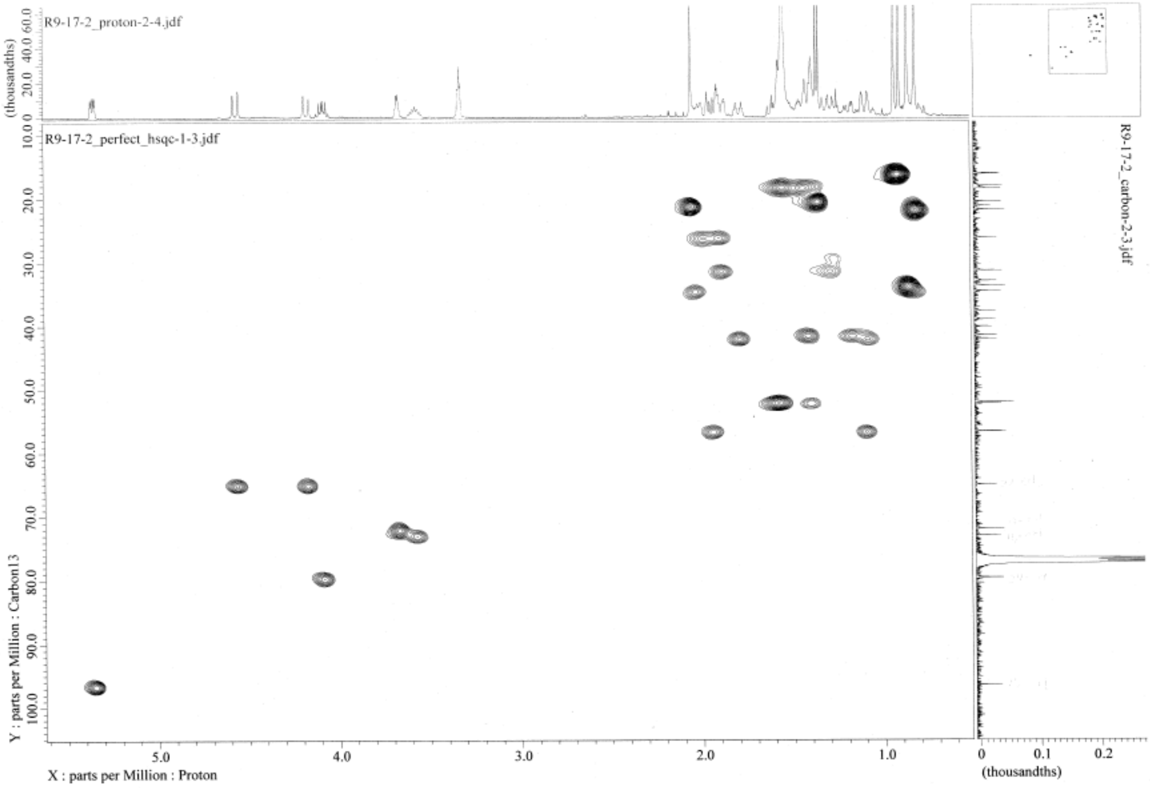

S29. HSQC spectrum of compound **4** in $CDCl_3$.

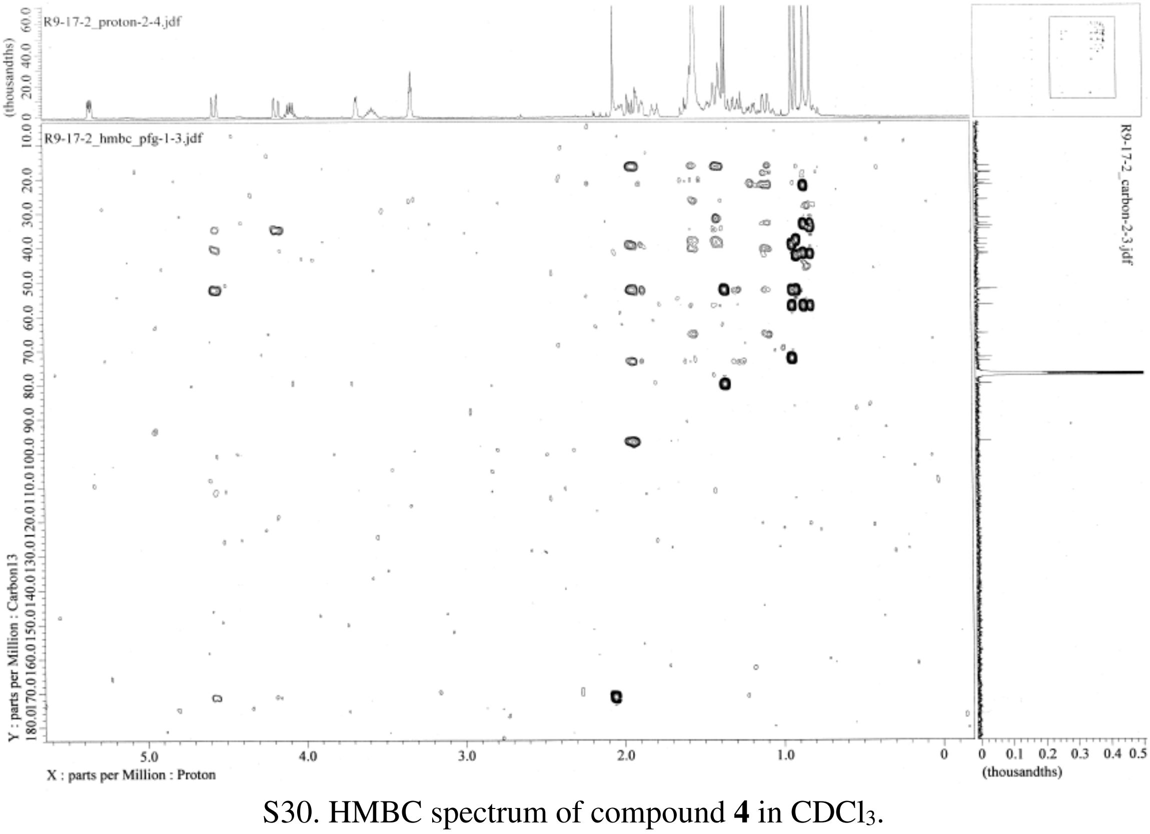

S30. HMBC spectrum of compound **4** in $CDCl_3$.

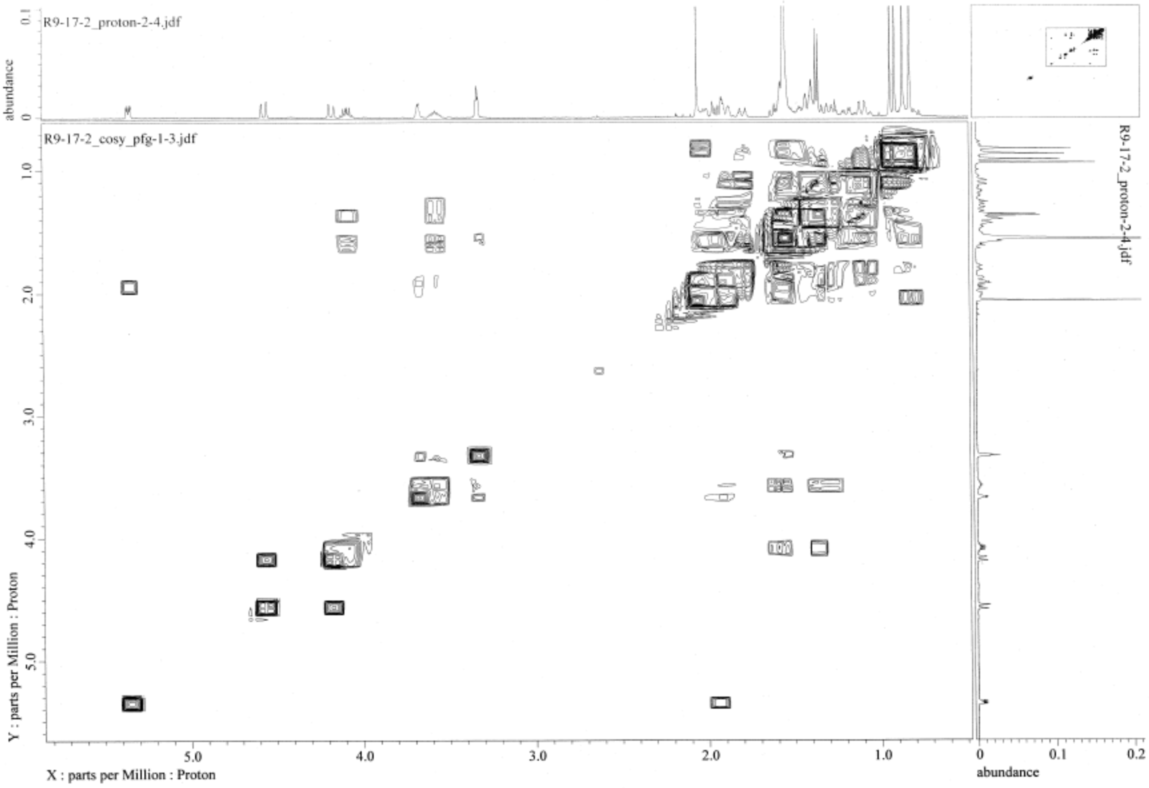

S31. ^1H-^1H COSY spectrum of compound **4** in $CDCl_3$.

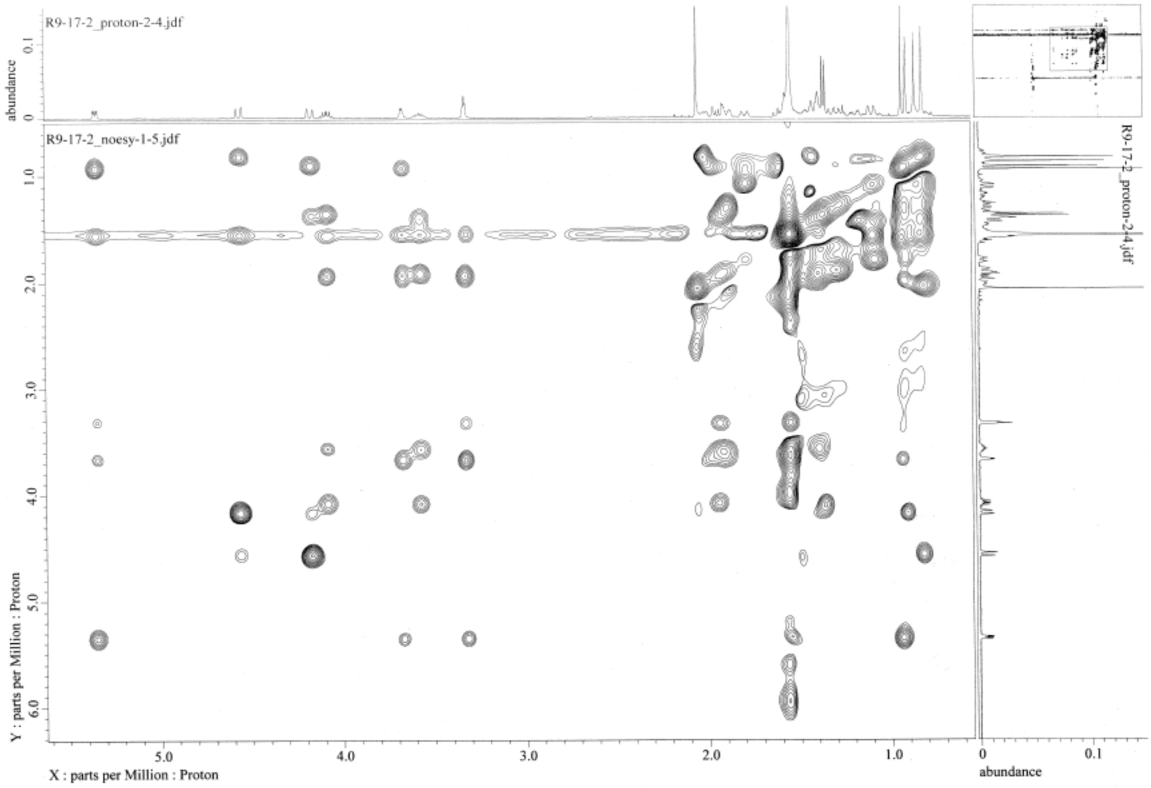

S32. NOESY spectrum of compound **4** in $CDCl_3$

Mass Spectrum SmartFormula Report

Analysis Info

Analysis Name	D:\Data\a2\R947_000005.d	2/20/2020 4:22:20 PM
Method	broadband first signal	Operator: YU HSIAO-CHING
Sample Name	R-9-4-7	Instrument: BRUKER FT-MS solariX
Comment	ESI Positive	

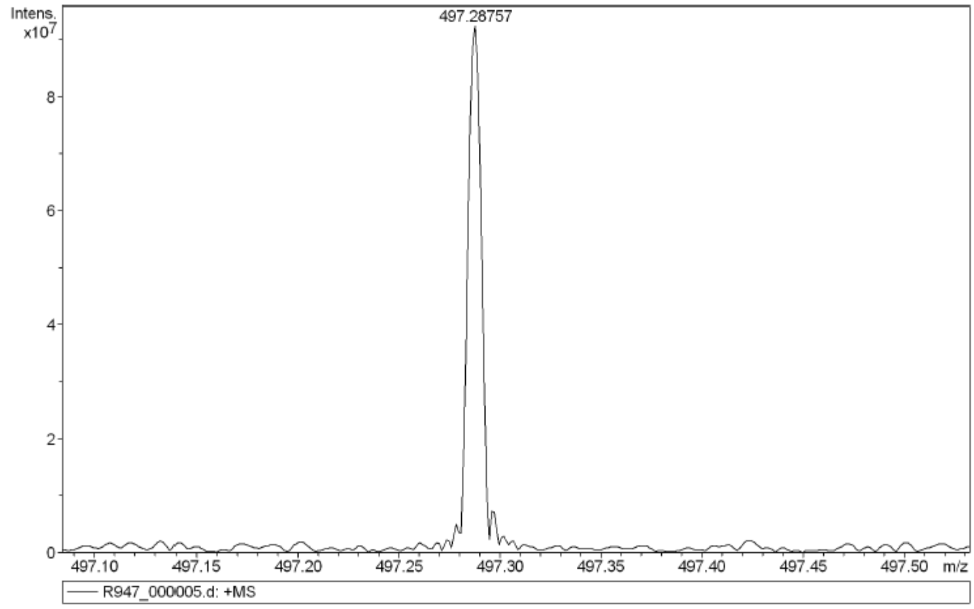

Meas. m/z	#	Formula	Score	m/z	err [mDa]	err [ppm]	mSigma	rdb	e⁻ Conf	N-Rule
497.28757	1	C 28 H 42 Na O 6	100.00	497.28736	-0.21	-0.42	10.0	7.5	even	ok

S33. HRESIMS spectrum of compound **5**.

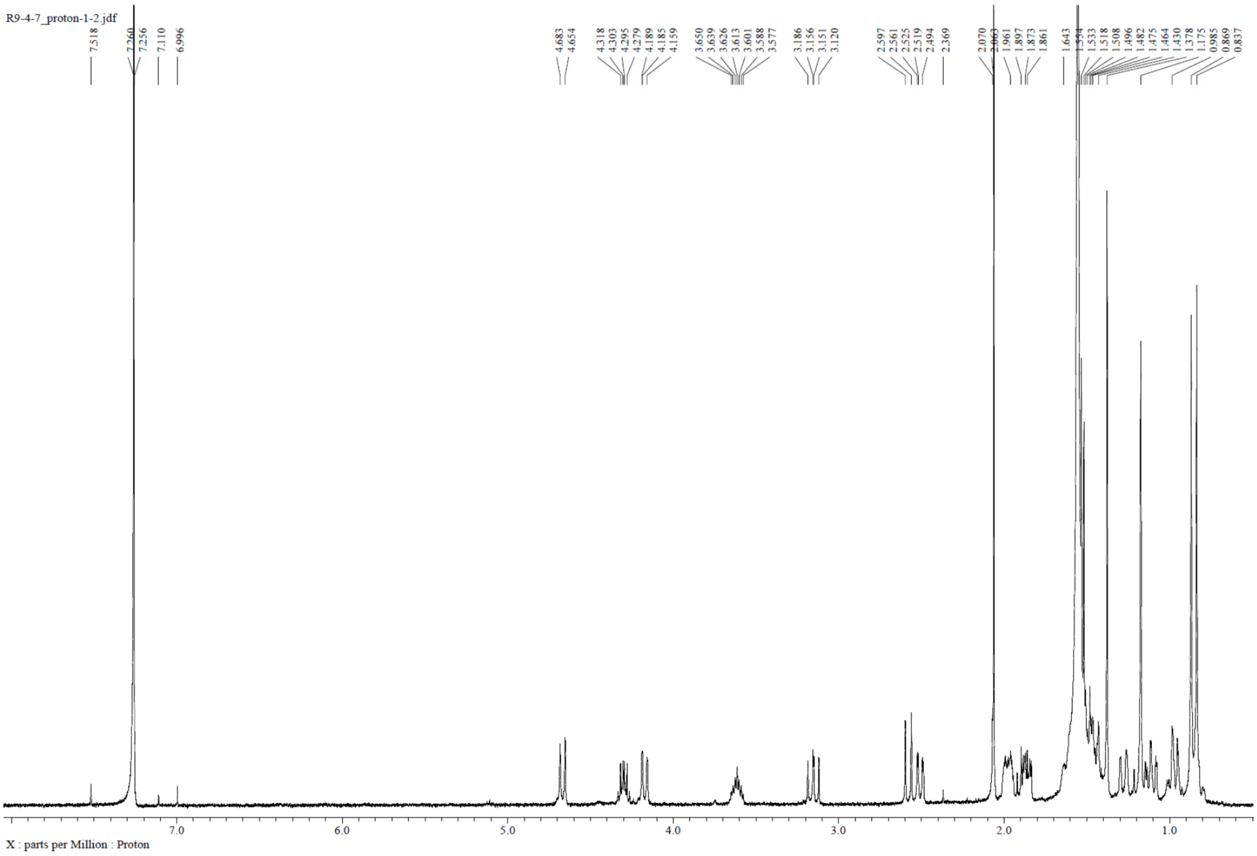

S34. ^{1}H NMR spectrum (400 MHz) of compound **5** in $CDCl_3$.

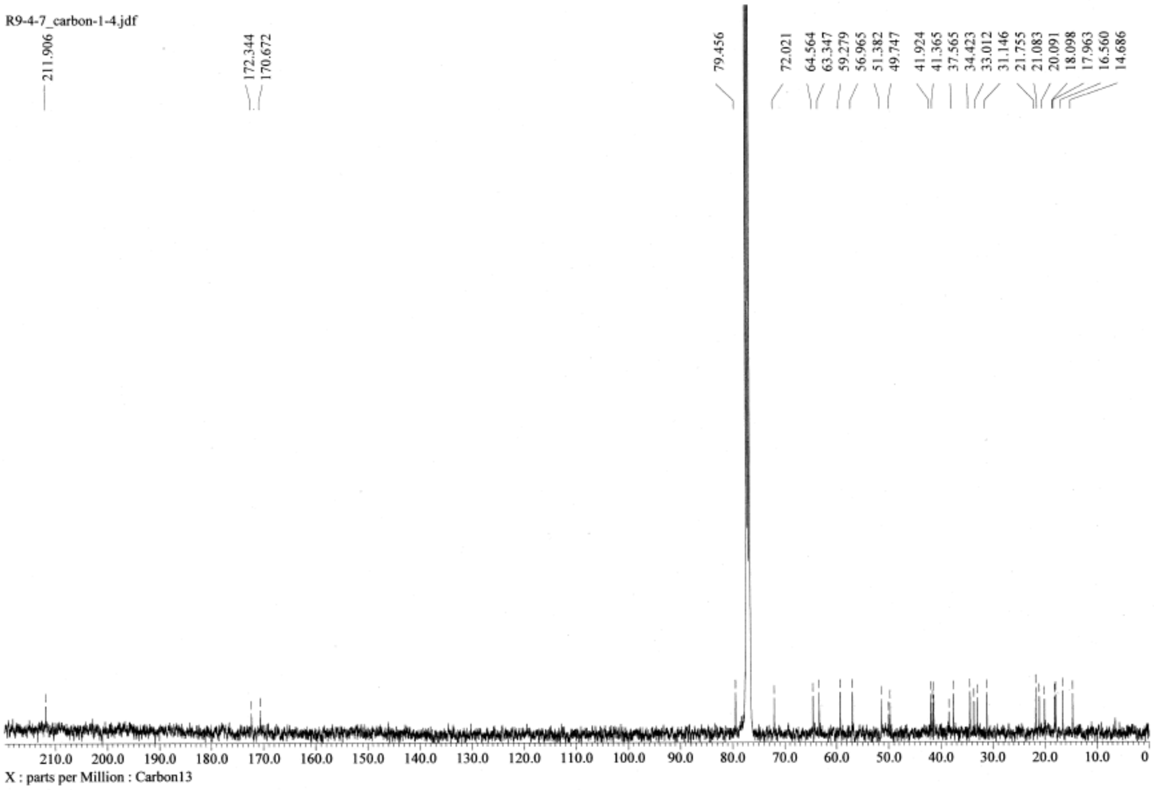

S35. ^{13}C NMR spectrum (100 MHz) of compound **5** in $CDCl_3$.

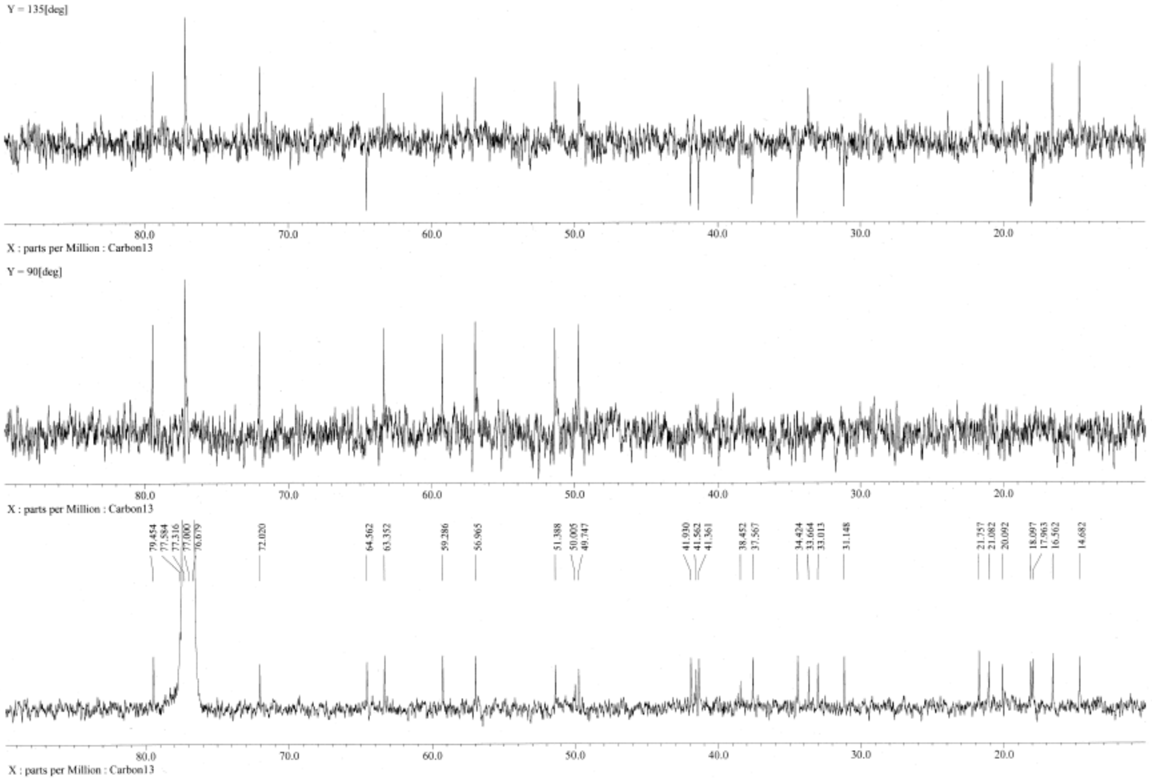

S36. DEPT spectrum (100 MHz) of compound **5** in $CDCl_3$.

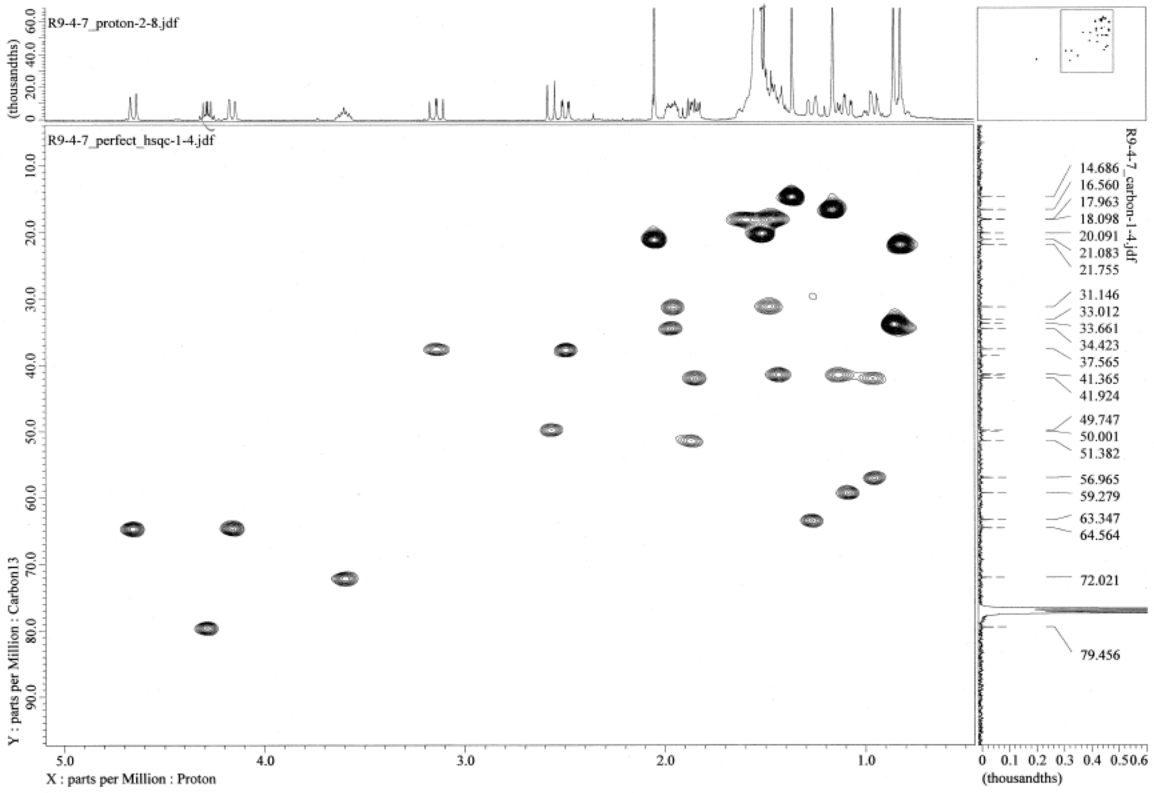

S37. HSQC spectrum of compound **5** in $CDCl_3$.

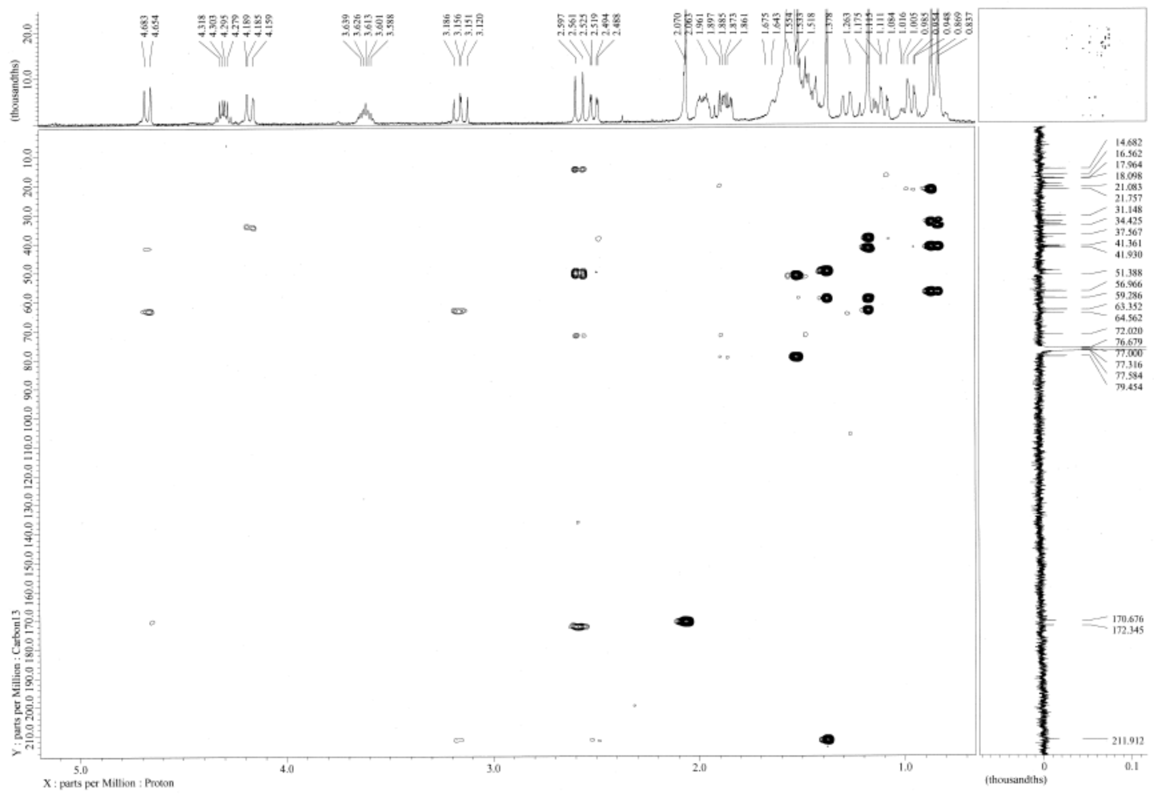

S38. HMBC spectrum of compound **5** in $CDCl_3$.

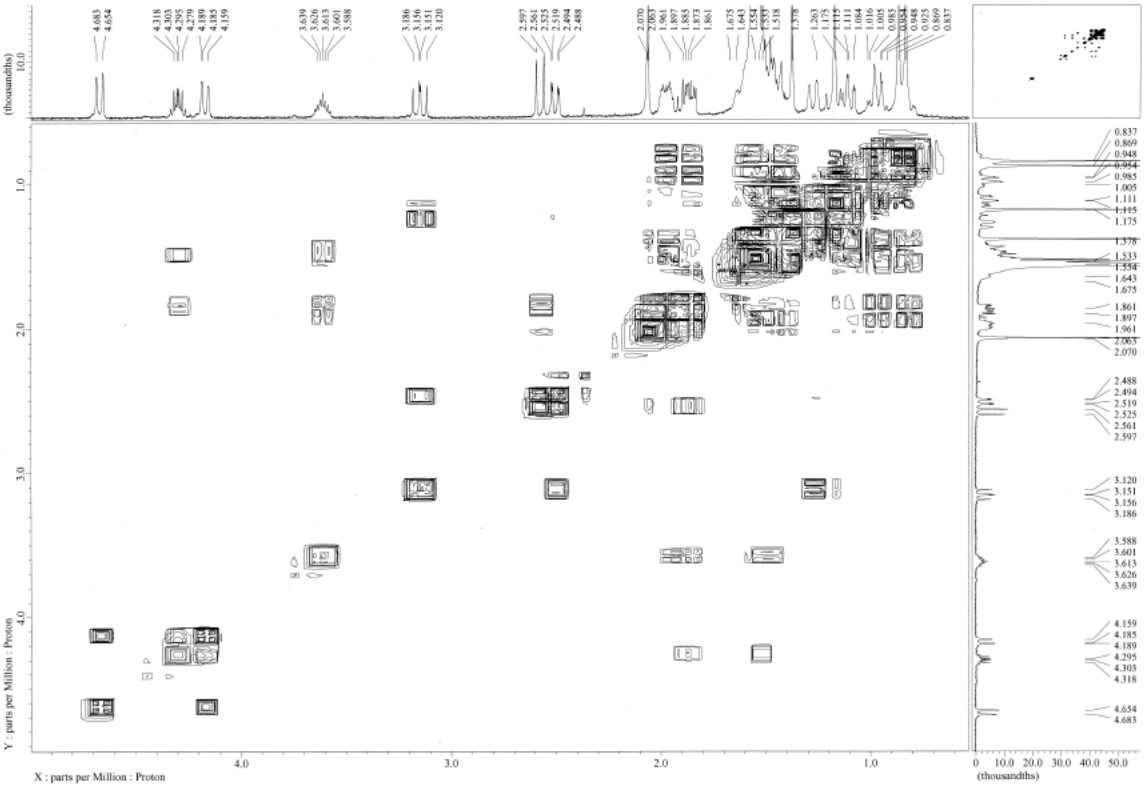

S39. ^1H-^1H COSY spectrum of compound **5** in $CDCl_3$.

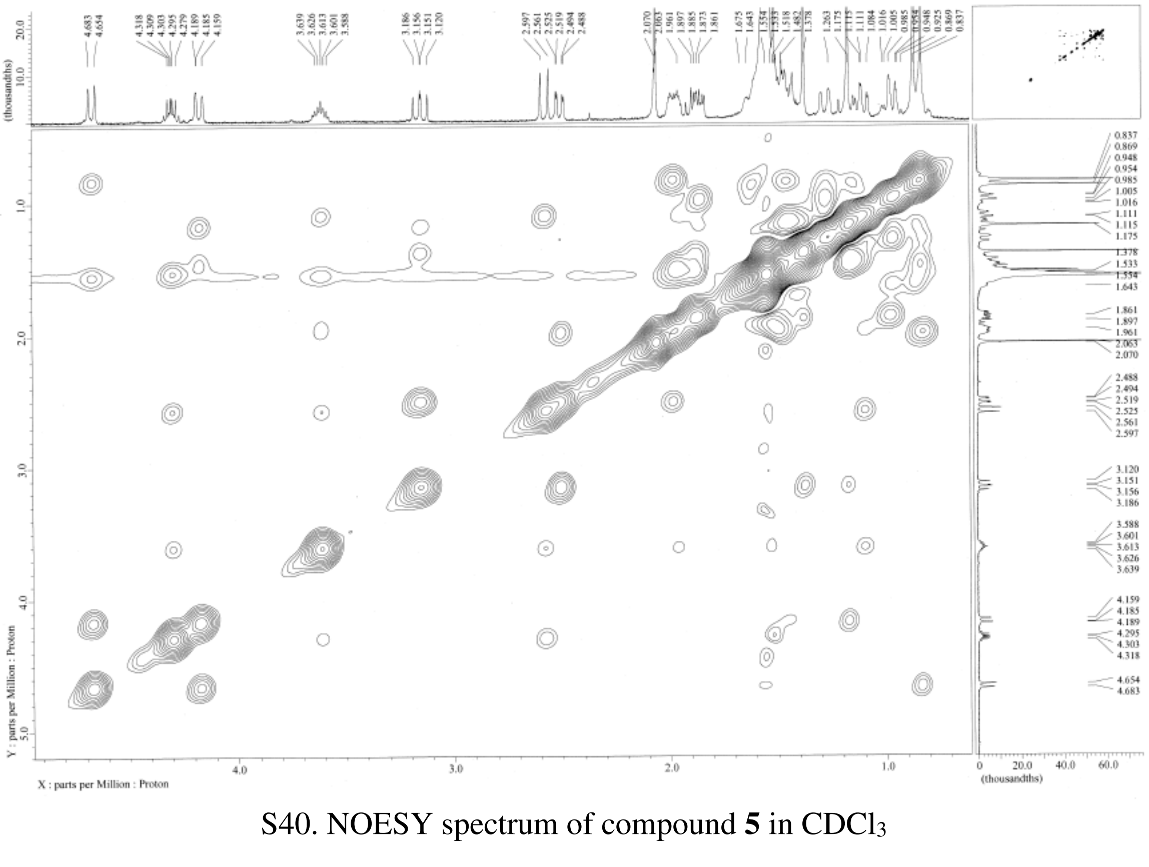

S40. NOESY spectrum of compound **5** in $CDCl_3$

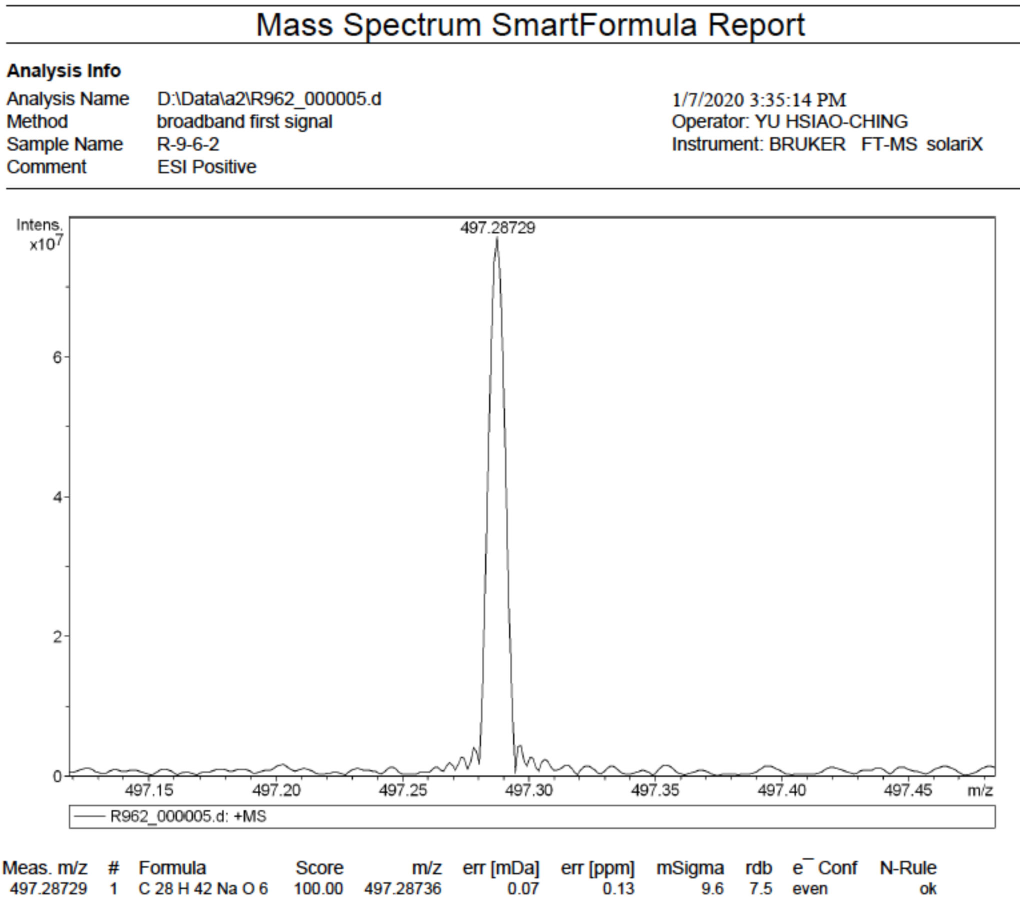

S41. HRESIMS spectrum of compound **6**.

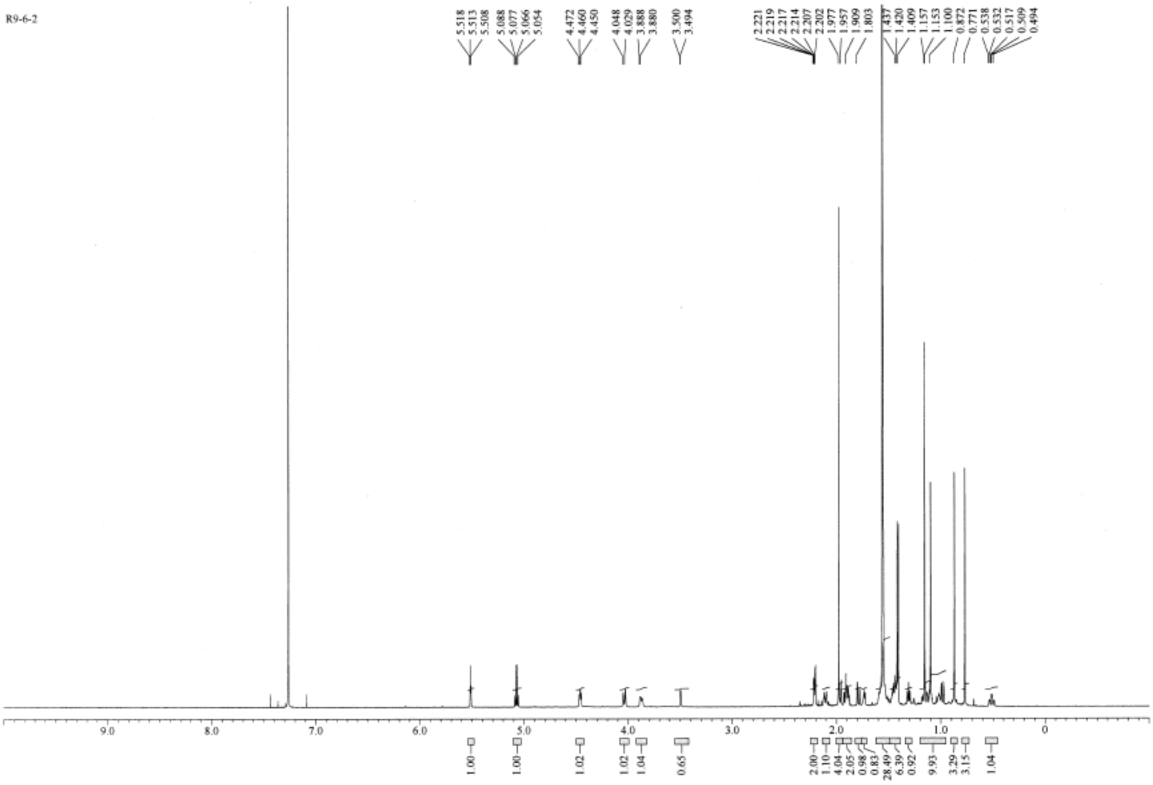

S42. ^{1}H NMR spectrum (600 MHz) of compound **6** in $CDCl_3$.

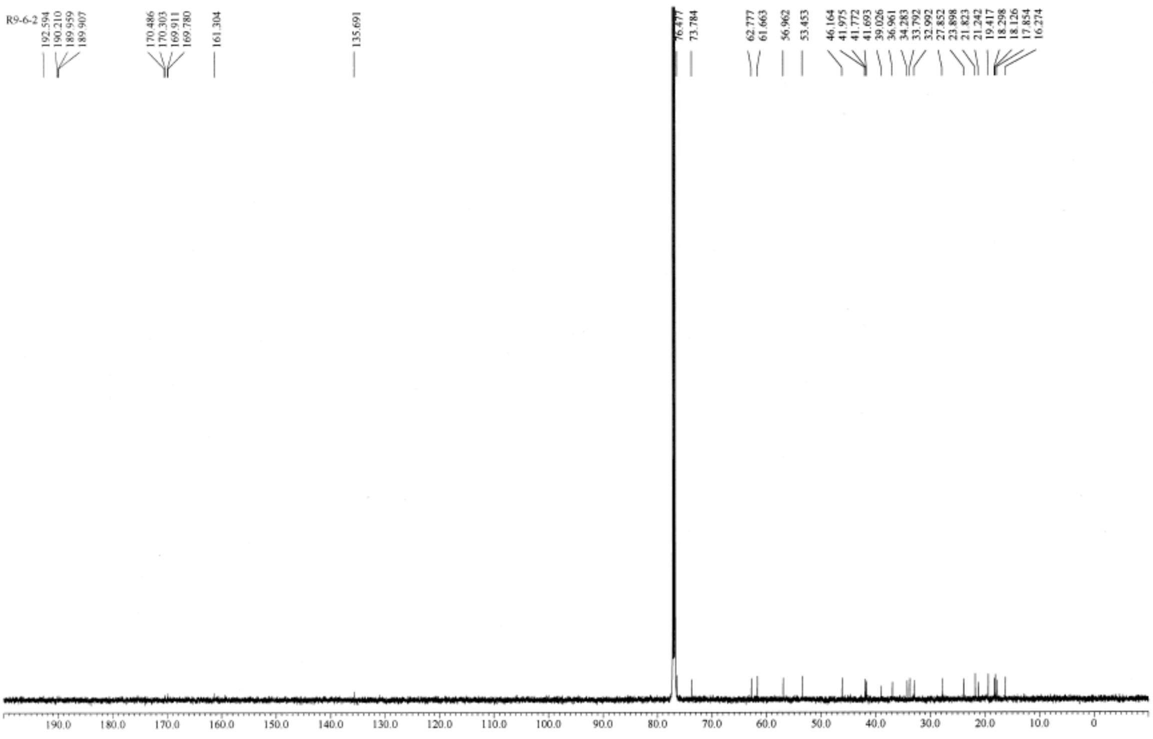

S43. ^{13}C NMR spectrum (150 MHz) of compound **6** in $CDCl_3$.

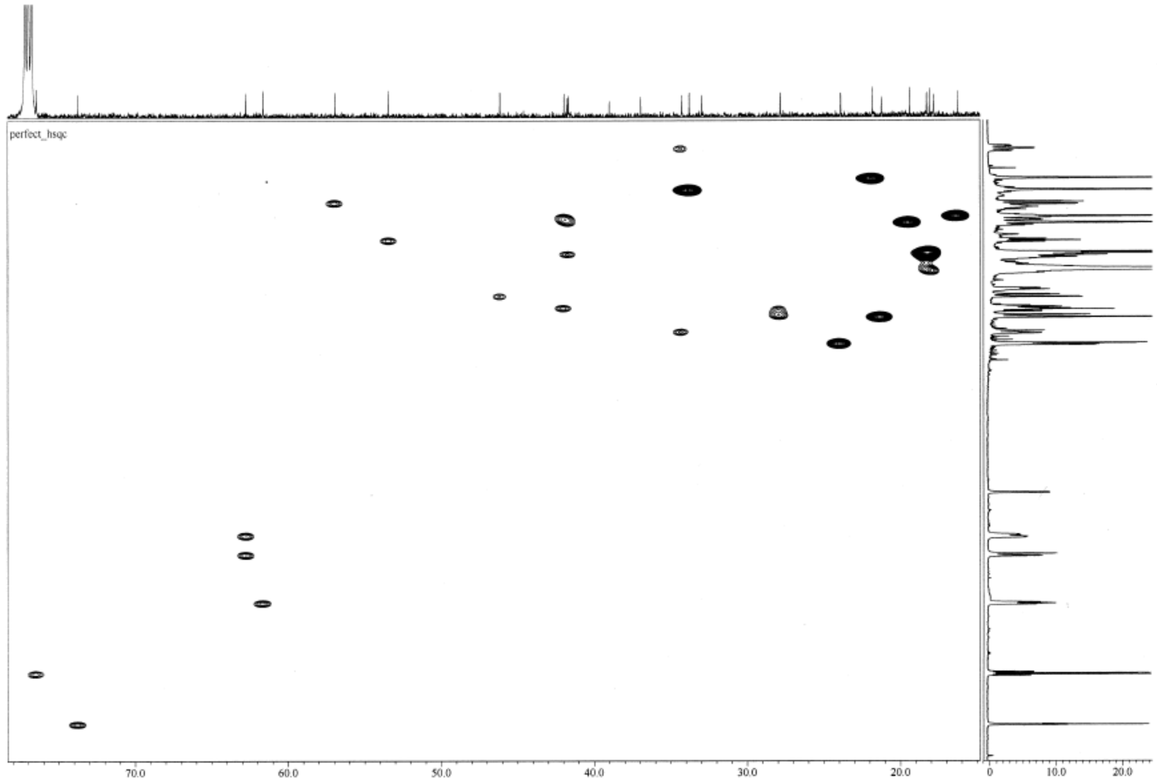

S44. HSQC spectrum of compound **6** in $CDCl_3$.

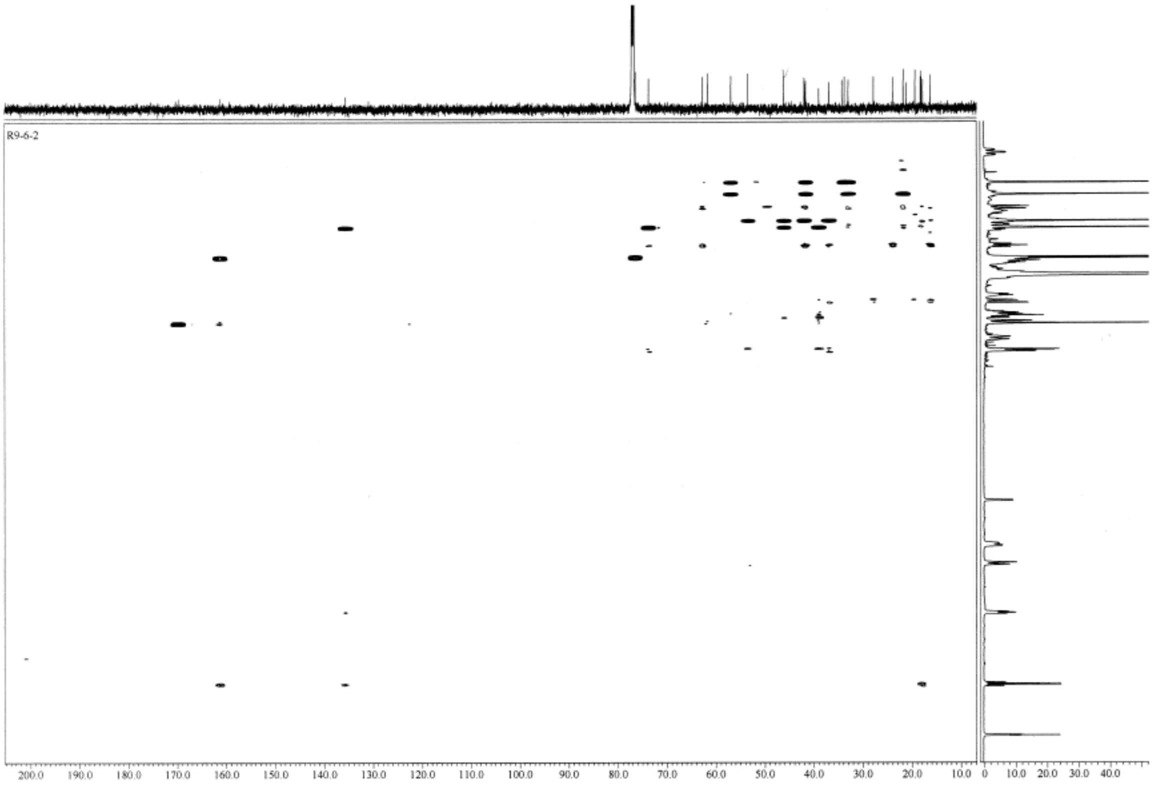

S45. HMBC spectrum of compound **6** in $CDCl_3$.

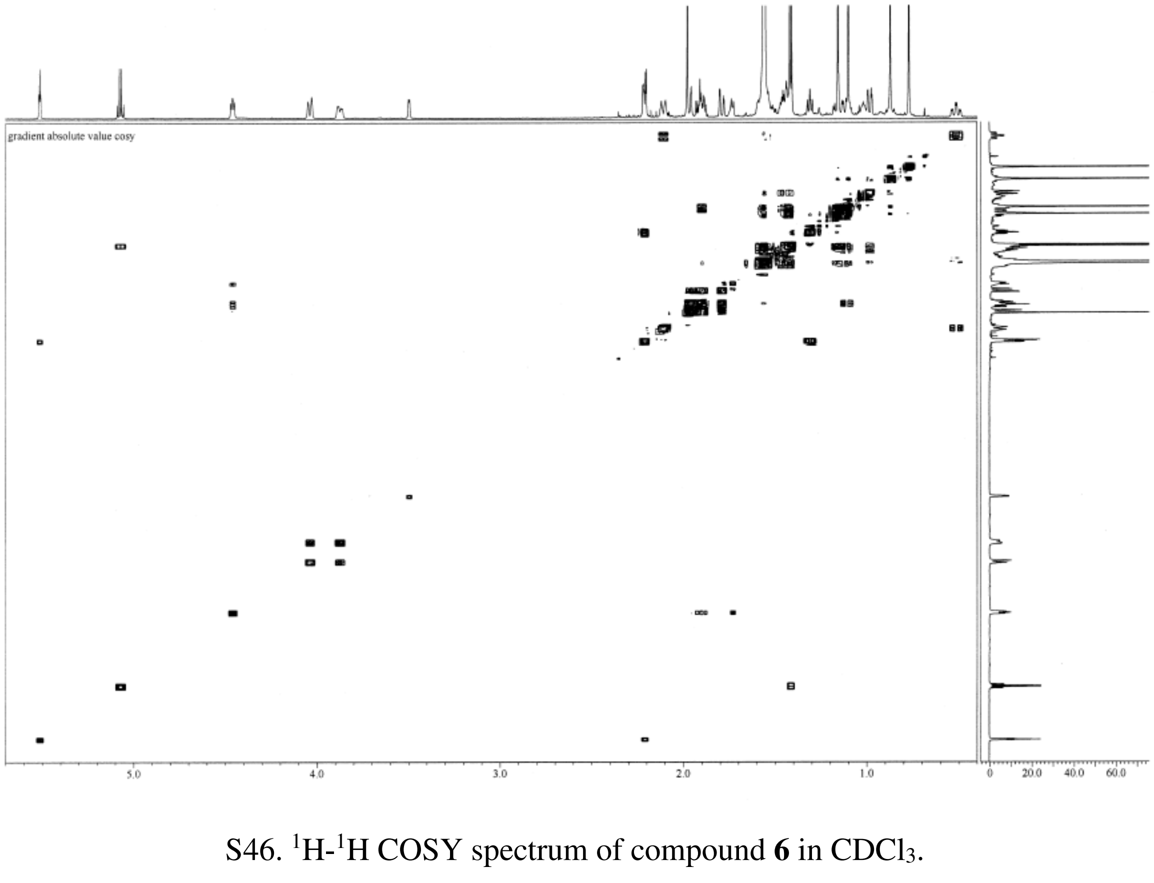

S46. ^{1}H-^{1}H COSY spectrum of compound **6** in $CDCl_3$.

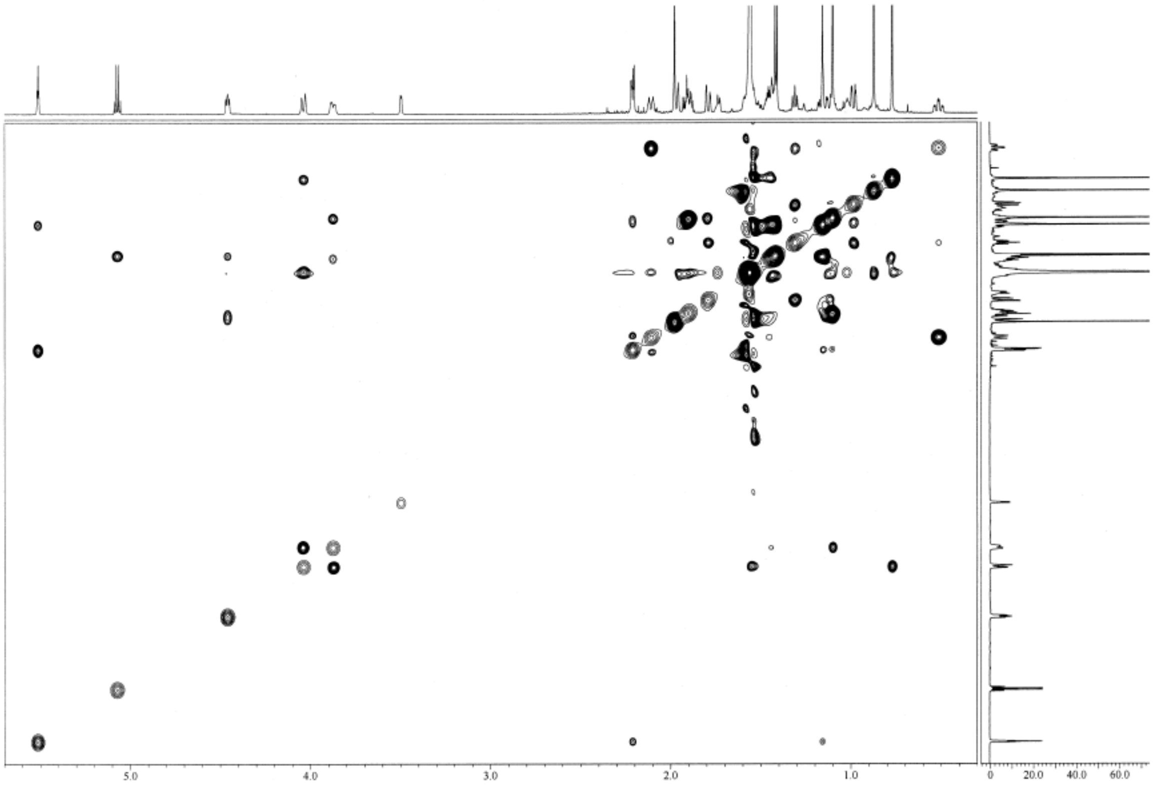

S47. NOESY spectrum of compound **6** in $CDCl_3$
